# Recapitulation-like developmental transitions of chromatin accessibility in vertebrates

**DOI:** 10.1186/s40851-019-0148-9

**Published:** 2019-11-14

**Authors:** Masahiro Uesaka, Shigeru Kuratani, Hiroyuki Takeda, Naoki Irie

**Affiliations:** 1 Department of Biological Sciences, The University of Tokyo, Tokyo, Japan; 2 Laboratory for Evolutionary Morphology, RIKEN Center for Biosystems Dynamics Research (BDR), Kobe, Japan; 3 Evolutionary Morphology Laboratory, RIKEN Cluster for Pioneering Research (CPR), Kobe, Japan; 4 Universal Biology Institute, The University of Tokyo, Tokyo, Japan

**Keywords:** Evolution, Development, Recapitulation, Parallelism, Gene regulatory evolution, ATAC-seq, Developmental hourglass model

## Abstract

The relationship between development and evolution has been a central theme in evolutionary developmental biology. Across the vertebrates, the most highly conserved gene expression profiles are found at mid-embryonic, organogenesis stages, whereas those at earlier and later stages are more diverged. This hourglass-like pattern of divergence does not necessarily rule out the possibility that gene expression profiles that are more evolutionarily derived appear at later stages of development; however, no molecular-level evidence of such a phenomenon has been reported. To address this issue, we compared putative gene regulatory elements among different species within a phylum. We made a genome-wide assessment of accessible chromatin regions throughout embryogenesis in three vertebrate species (mouse, chicken, and medaka) and estimated the evolutionary ages of these regions to define their evolutionary origins on the phylogenetic tree. In all the three species, we found that genomic regions tend to become accessible in an order that parallels their phylogenetic history, with evolutionarily newer gene regulations activated at later developmental stages. This tendency was restricted only after the mid-embryonic, phylotypic periods. Our results imply a phylogenetic hierarchy of putative regulatory regions, in which their activation parallels the phylogenetic order of their appearance. One evolutionary mechanism that may explain this phenomenon is that newly introduced regulatory elements are more likely to survive if activated at later stages of embryogenesis. Possible relationships between this phenomenon and the so-called recapitulation are discussed.

## Background

Animal embryogenesis generally proceeds from a simple, single-celled zygote to a complex, multicellular organism. This has led some biologists to propose a parallelism between development and evolution (or phylogenetic classification) [1–3]. The recapitulation theory, for example, predicts that animal development proceeds along a phylogenetic pathway, sequentially developing from features that are more ancestral to those that are more derived. Recent transcriptome-based studies, however, have not supported such recapitulation in vertebrate embryogenesis [4–8]. Instead, such studies have found that the most highly conserved patterns of gene expression appear at mid-embryonic stages, during which organogenesis occurs, with divergent profiles found at earlier and later stages (the developmental hourglass model [9]). Nevertheless, the development of some morphological features in the post-phylotypic period, such as loss of limbs in snakes and whales (hindlimbs) [10, 11] and jaw development in needlefishes [12], do apparently follow the recapitulative pathway of their evolution. Turtle embryogenesis also passes through several anatomical patterns resembling those of ancestral fossils [13]; modern turtles first develop scapula anlagen dorsal to the rib cage (a component of the turtle carapace), as occurs in turtle ancestors and other amniotes. Only subsequently does the scapula tilt and relocate ventral to the rib cage, producing the anatomy typical of modern turtles [13]. One possible reconciliation of these ideas is that embryogenesis mirrors the phylogenetic trajectory (palingenesis), recapitulating its evolutionary history from the conserved mid-embryonic stages (the “phylotypic period” in vertebrates [9, 14, 15]) [16].

Previous studies based on the expression profiling of protein-coding genes during embryogenesis have tried to detect potential parallelisms between development and evolution, such as possible shifts of the conserved mid-embryonic stages to later stages when analyzed at smaller evolutionary scales [4, 7, 8]. However, none of these studies detected later recapitulative patterns in mid-to-late embryogenesis [4–8], instead supporting persistent conservation of the mid-embryonic stages [8, 17, 18]. Nevertheless, it should be noted that repeated recruitment of the same protein-coding genes at different developmental stages [8] would obscure any recapitulative pattern, which highlights the importance of using alternative experimental approaches to examine evolutionary changes in gene expression regulation that occurred along a phylogenetic trajectory.

Given that the majority of genetic changes associated with the emergence of major vertebrate groups are concentrated in potential regulatory regions, rather than protein-coding regions [19, 20], it would be useful to measure the activity of gene regulatory regions throughout embryogenesis and to trace their evolutionary origins simultaneously. One previous study focused on the activation of enhancers during mouse embryogenesis; however, it did not evaluate whether the temporal activation patterns of regulatory regions paralleled their evolutionary ages, finding only that enhancers activated during the conserved mid-embryonic stages are also conserved at the sequence level [21].

In the present study, we hypothesized that more recently acquired regulatory regions tend to be activated sequentially in mid-to-late embryogenesis. We tested this hypothesis by focusing on active regulatory regions in embryos and their evolutionary ages in three vertebrate species.

## Results

### Genome-wide mapping of accessible chromatin regions during vertebrate embryogenesis

**Fig. 1:**
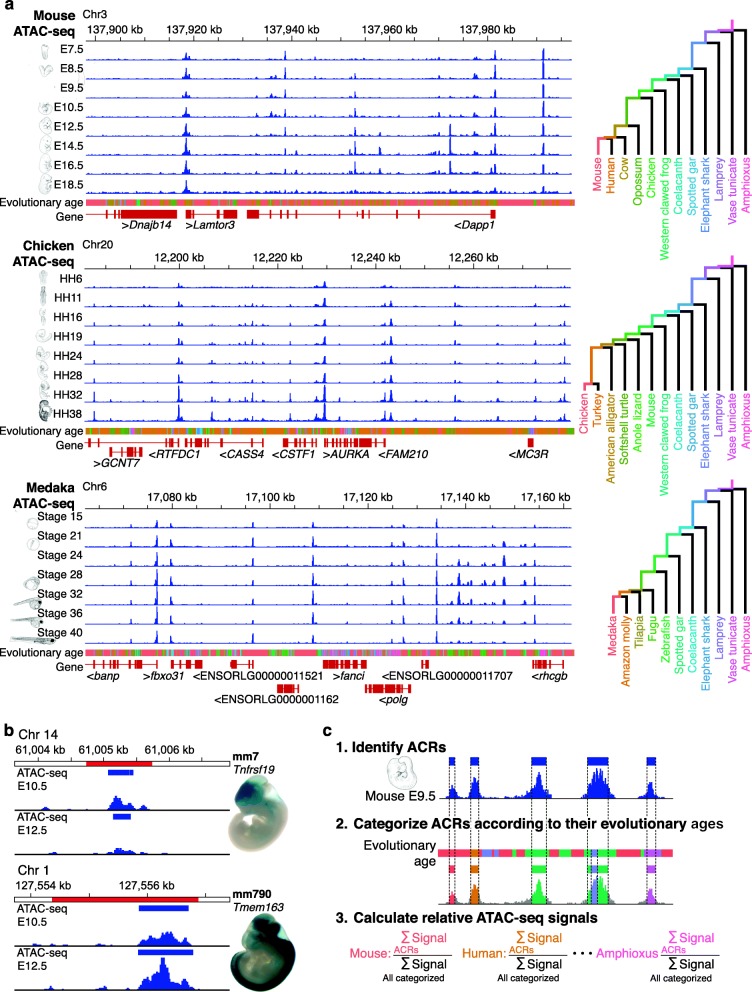
Strategy for assessing accessible chromatin landscapes in vertebrate embryos. **a** Genome browser views showing enrichment of whole-embryo ATAC-seq reads in representative regions of mouse, chicken, and medaka genomes, respectively. ATAC-seq read enrichment is presented as the mean of three biological replicates. Colors below the read enrichment represent the estimated evolutionary ages of genomic regions that correspond to the tracks of the evolutionary trajectories, which are shown as a phylogenetic tree on the right. **b** The representative ACRs (blue boxes) overlapping with annotated enhancers (red regions) acquired from the VISTA Enhancer Database [23]. For each enhancer, ATAC-seq read enrichment in E10.5 and E12.5 mice and in vivo enhancer activity in E11.5 mice are shown with the VISTA Enhancer ID, the flanking gene, and an embryo image from the VISTA Enhancer Database [23]. **c** Schematic diagram showing the three steps for estimating the relative ATAC-seq signal for each evolutionary age: (1) ACRs were identified by ATAC-seq signal intensity; (2) ACRs were categorized according to their estimated evolutionary ages; and (3) for each developmental stage, the percentage corresponding to the summed signal intensities of each evolutionary category divided by the total signal intensities for all evolutionary categories was calculated (relative ATAC-seq signals)

We collected mouse (*Mus musculus*), chicken (*Gallus gallus*), and medaka (*Oryzias latipes*) embryos at developmental stages covering the phylotypic period and later stages (Additional file 1: Table S1). For each species, the phylotypic period was estimated as a period around the stage that shows the highest cross-species similarity of whole-embryo transcriptomes (around E9.5 in mouse [6, 8], around HH16 in chicken [6–8], and around stage 24 in medaka; Additional file 2: Figure S1 and Additional file 3: Text S1). Regulatory regions active in whole embryos were then estimated by an assay for transposase-accessible chromatin using sequencing (ATAC-seq [22]; Fig. 1a), as accessible chromatin marks active regulatory regions, including enhancers, silencers, and promoters [24, 25].

We measured chromatin accessibility at different developmental stages in the three species by using ATAC-seq (Fig. 1a). For each developmental stage, we generated a genome-wide map of accessible chromatin regions (ACRs) and identified more than 150,000 ACRs as putative regulatory regions (Additional file 1: Table S2). Visualization of representative genomic regions indicated that our data robustly reflect changes in ATAC-seq read enrichments during embryogenesis (Fig. 1a). For example, the ATAC-seq reads were enriched at the transcription start sites (TSSs) of genes with low background noise, and the read enrichment profiles differed across developmental stages (Fig. 1a). To further validate our ATAC-seq data, we evaluated the signal-to-background ratio of the whole-embryo ATAC-seq data (Additional file 1: Table S3) and confirmed that the detected signal levels of chromatin accessibility over those from genomic background were sufficiently high. In all three species, the genomic distribution of ACRs was similar to that reported in previous studies [26, 27] (Additional file 2: Figure S2); ACRs were significantly enriched at promoters (two-sided Fisher’s exact test, *P* < 2.2 × 10^− 16^; Additional file 1: Table S4), with the majority mapped to intergenic regions or introns (Additional file 2: Figure S2). The mouse ACRs covered as much as 95.4% of the mouse enhancers registered in the VISTA Enhancer Database (Fig. 1b and Additional file 2: Figure S3), suggesting that the whole-embryo ATAC-seq sensitively estimates chromatin accessibility of the active regulatory regions in different parts of embryos. In addition, by focusing on representative enhancer regions, ATAC-seq signal intensities appeared to be stronger for enhancers that drive expression in larger cell populations (Additional file 2: Figure S3), consistent with the prediction that signal intensity reflects the number of cells in which the examined region is accessible. The ATAC-seq signal intensities at ACRs were robustly reproduced across the three biological replicates (Additional file 1: Table S5). These results collectively indicate that these whole-embryo ATAC-seq data provide a reliable accessible chromatin landscape and enable the quantitative estimation of chromatin accessibility in entire embryos at different developmental stages.

### Estimating the evolutionary ages of the ACRs

To infer the evolutionary ages of the genomic regions, we first generated pairwise alignments of different chordate genomes to the reference genomes for mouse, chicken, and medaka (Additional file 1: Table S6), and determined the species that share the corresponding sequences of the region. For each genomic region, evolutionary age was defined as the length of time from the most recent common ancestor of all the species that share the sequence (Fig. 1a). The ACRs were categorized according to the estimated evolutionary ages (see Methods for details). For ACRs that consist of sequences with multiple evolutionary origins (~ 40%), we subdivided them into separate ACRs by different evolutionary origins. We first focused on strictly conserved ACRs shared by all the species in certain monophyletic groups, but not found in any outgroup species (this excludes ACRs that were secondarily lost in any of the species in the group; method I in Additional file 2: Figure S4a, b).

**Fig. 2:**
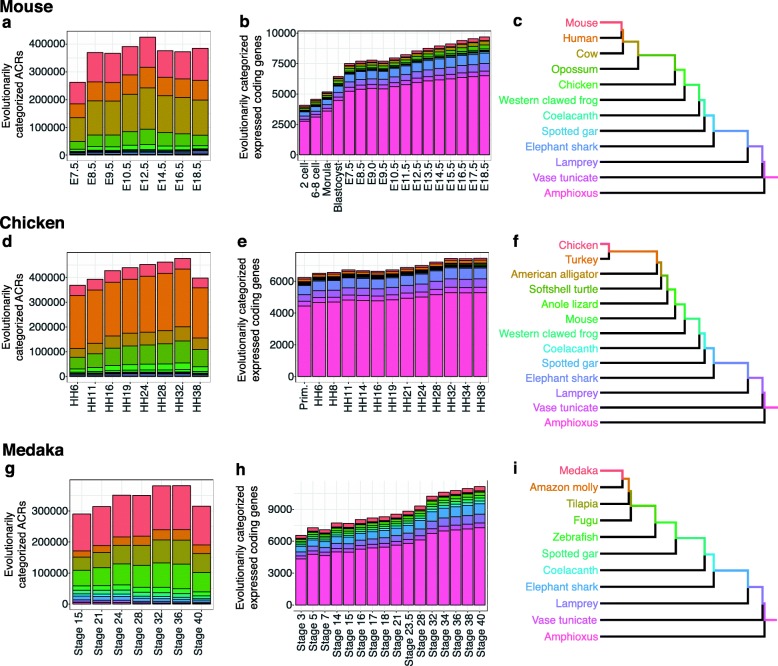
Numbers of ACRs and expressed protein-coding genes categorized according to evolutionary ages. Stacked bar graphs show the numbers of evolutionarily categorized ACRs (**a**, **d**, **g**) and expressed (FPKM > 1) protein-coding genes (**b**, **e**, **h**) at each developmental stage in mouse (**a**, **b**), chicken (**d**, **e**), and medaka (**g**, **h**). Evolutionary ages of ACRs were estimated based on Method I (for details, see Methods and Additional file 2: Figure S4). The evolutionary ages of protein-coding genes were estimated according to the most recent common ancestors of all the species sharing the homologs; the expressed genes that were estimated to be lost secondarily in any of the compared species were excluded (see Methods for details). Colors in each stacked bar graph indicate the categories of the evolutionary ages of each element. Each evolutionary category includes ACRs or expressed protein-coding genes that originated during the correspondingly colored period in the phylogenetic trees shown in **c**, **f**, and **i**

We also estimated evolutionary ages using three alternative methodologies (Additional file 2: Figure S4a, b). In brief, method II included not only strictly conserved ACRs (as in the method I), but also those presumably lost secondarily (10–20% of all ACRs). Method III also focused on strictly conserved ACRs, but each identified ACR was considered as a single regulatory element with a single evolutionary age (defined by the evolutionary ages of sequences comprising more than one-third of the ACR; Additional file 2: Figure S4a, b). Finally, method IV covered secondarily lost sequences without subdividing ACRs by multiple evolutionary ages (Additional file 2: Figure S4a, b). We confirmed that all four methods yielded closely similar distributions of evolutionary ages for ACRs (Fig. 2a, d, g; Additional file 2: Figure S5a, c, e).

As expected, while less than 5% of ACRs were found to be evolutionarily older than the vertebrate–urochordate split (Fig. 2a, d, g and Additional file 2: Figure S5a, c, e), those in protein-coding genes represented more than two-thirds of the total (Fig. 2b, e, h and Additional file 2: Figure S5b, d, f). This is consistent with previous reports [19, 20], that non-coding regions are much more diverse than protein-coding genes, and suggests that analysis of ACRs may reveal higher evolutionary resolution than comparisons of gene expression profiles in vertebrate development.

### Recapitulative pattern observed for chromatin accessibility after the phylotypic period

To test whether evolutionarily newer genomic regions tend to become sequentially accessible as embryogenesis proceeds, we analyzed whole-embryo temporal patterns of ACRs and their evolutionary ages. In measuring chromatin accessibility, we took into account the signal intensity of ATAC-seq, as this may reflect fractions of cells in an embryo, which would thus help test the overall tendency of a recapitulative pattern at the whole-embryo level. Briefly, we summed the ATAC-seq signal intensities of ACRs of the same evolutionary age, and calculated their percentage against the total signal intensities of all ACRs (Relative ATAC-seq signals; Fig. 1c).

**Fig. 3:**
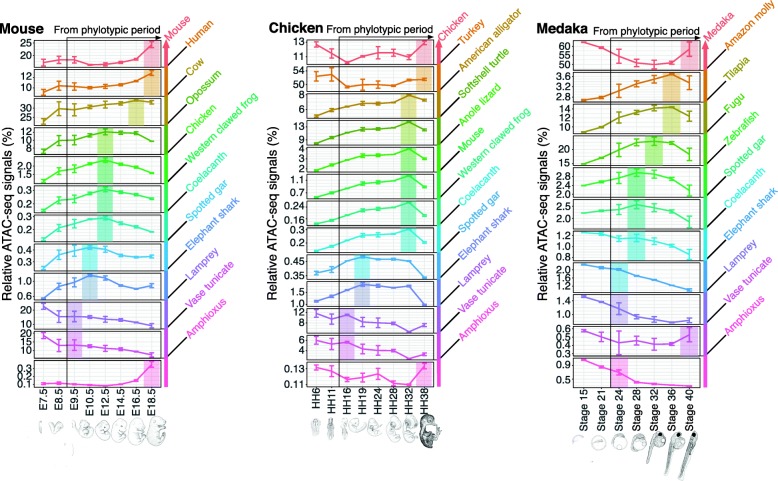
Transition of developmental stages with the maximum evolutionarily categorized chromatin accessibility during vertebrate embryogenesis. For each developmental stage in three vertebrate species (mouse, chicken, and medaka), the percentages on the vertical axis represent the summed signal intensity for each evolutionary category of ACRs divided by the total signal intensity for all categories (relative ATAC-seq signal). The color of each category indicates the estimated evolutionary age of the region (shown at right). In each graph, the developmental stages with the highest signal from the potential phylotypic period are highlighted in the corresponding colors, as is the range that showed a recapitulative pattern for unknown reasons. Error bars indicate the standard deviation of three biological replicates for each developmental stage. Changes in the relative ATAC-seq signals were statistically significant (Kruskal–Wallis rank sum test) in all cases, except for the vase tunicate category in medaka. Detailed statistical information is provided in Additional file 1: Table S7

By analyzing these relative ATAC-seq signals in the mouse, chicken, and medaka, we found that maximum signals of ACRs with younger evolutionary ages tended to emerge at later developmental stages (from E9.5 in mouse, HH16 in chicken, and stage 24 in medaka; Fig. 3). In mouse, for example, the relative ATAC-seq signals of the youngest and second youngest evolutionary categories (i.e., mouse-specific ACRs and mouse–human clade-specific ACRs) were highest at the latest developmental stage (E18.5). For the next youngest category (ACRs specific to the mouse–human–opossum clade), the signal peak was found at the second latest developmental stage (E16.5). Sequences of similar transitions were observed in all the examined species. In the meanwhile, the distribution of signal peaks along developmental stages differed by species, showing different degrees of steepness of the recapitulative pattern (Fig. 3). For example, stage HH32 chicken embryo showed the highest signals in six successive evolutionary categories, whereas no stage showing a steep transition in the recapitulative pattern was detected in the other species. Nonetheless, the phenomenon in which evolutionarily newer ACRs tend to become accessible at later developmental stages was consistently observed in all three species examined. This recapitulative tendency was especially pronounced after the phylotypic period, which was also consistently observed in all the species analyzed (Fig. 3). These results suggest that, from the mid-embryonic stages onward, developmental whole-embryo chromatin accessibility parallels the evolutionary ages of the putative regulatory regions in a recapitulative pattern.

**Fig. 4:**
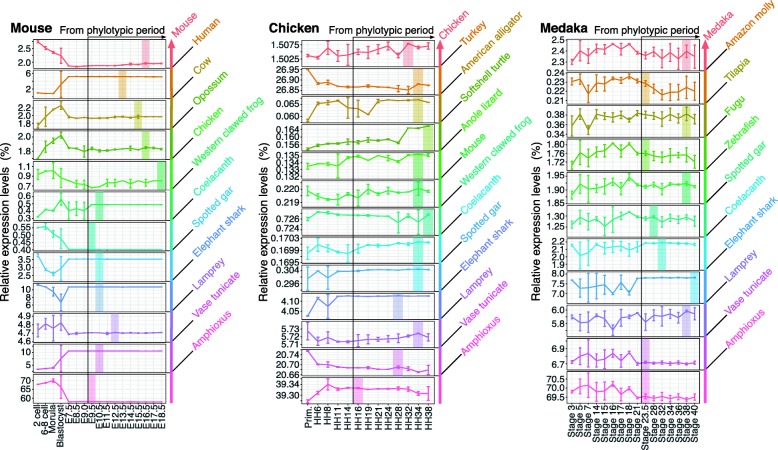
No recapitulative pattern was observed in developmental gene expression levels For each developmental stage, summed expression levels of evolutionarily categorized protein-coding genes were shown as the percentage relative to the total expression levels of all evolutionary categories. The color of each category corresponds to the estimated evolutionary age of the protein-coding genes (shown at right). The evolutionary age of each protein-coding gene was estimated according to the most recent common ancestors of all the species sharing the homologs. Genes that were estimated to be secondarily lost in any of the compared species were excluded (see Methods for details). In each graph, developmental stages with the highest value after the potential phylotypic period are highlighted in the corresponding color. Error bars indicate the standard deviation of biological replicates for each developmental stage. Statistical information for the Kruskal–Wallis rank sum test is given in Additional file 1: Table S8.

A potential caveat is that these analyses took ATAC-seq signal intensities into account, rather than simply measuring the relative sequence lengths of ACRs within the genome. We therefore performed an alternative analysis based on sequence lengths of ACRs, rather than on ATAC-seq signal intensities (Additional file 2: Figure S6). In brief, we summed the sequence length of ACRs having the same evolutionary age and calculated the percentage of the total length of all ACRs. The results of this analysis showed essentially the same pattern as those obtained with ATAC-seq signal intensities, suggesting that genomic repertoires of active regulatory regions also show recapitulative patterns during development. A similar analysis of coding gene expression profiles did not show any sign of recapitulation (Fig. 4 and Additional file 2: Figure S7), as had been suggested by previous reports [7, 8]. This may be attributed to a lower resolution of the approach based on gene expression, since protein-coding genes are repeatedly recruited during embryogenesis in different regulatory contexts.

Another potential bias in the interpretation of the present results could arise from how we defined evolutionary age. To ensure the observed recapitulative tendency in chromatin accessibility is robust against how we defined the evolutionary ages of ACRs, we performed analyses using different methods to estimate the evolutionary ages of ACRs (Additional file 2: Figure S8 and Additional file 3: Text S2.1) and ones with a different genome set including more evolutionary distant species for phylogenetic comparison (Additional file 2: Figure S9 and Additional file 3: Text S2.2). In these analyses, the recapitulative pattern was robustly observed (Additional file 2: Figures S8 and S9). Additionally, we confirmed that analyses with different criteria in filtering ATAC-seq reads (with only uniquely aligned reads; without ATAC-seq reads aligned to the mitochondrial genome; without read-depth normalization) consistently reproduced closely similar recapitulative patterns (Additional file 2: Figure S10 and Additional file 3: Texts S2.3–2.5). Our results thus indicate that the observed recapitulative pattern is robustly reproducible independent of the analytical conditions or datasets used.

Meanwhile, evolutionarily older ACRs did not follow the recapitulative pattern in chromatin accessibility in any of the three vertebrates studied (Fig. 3). For example, in mouse, the highest ATAC-seq signal for the olfactores–cephalochordate split was not at earlier stages, but rather at the latest developmental stage (Fig. 3). Non-recapitulative patterns were also observed in other species, and the older evolutionary age limit for a recapitulative pattern differed by species and analytical conditions (Fig. 3 and Additional file 2: Figures S8–10). In contrast, at least for ACRs newer than the gnathostome–cyclostome split, the recapitulative pattern was consistent among all the analytical conditions tested (Fig. 3 and Additional file 2: Figures S8–10). The reason for this boundary is unclear. Further detailed study involving more species may help to clarify whether the gnathostome–cyclostome split is the oldest boundary for the recapitulative pattern.

Before the phylotypic period, no recapitulative tendency was apparent; in chicken and medaka, the highest ATAC-seq signals of evolutionarily new ACRs appeared at pre-phylotypic stages (Fig. 3). In the mouse, the recapitulative pattern was observed at stages before the phylotypic period (E7.5 to E18.5; Fig. 3). Additional analysis with a publicly available dataset [28] showed that the highest signal for the evolutionarily newest category was detected at the 8-cell stage in mouse, indicating that the recapitulative pattern is not expected before E7.5 (Additional file 2: Figure S11), which may not conflict with the phylotypic period (E9.5). Taken together, our data indicate that the recapitulation-like pattern would be observed as a sequence of regulatory activities after the phylotypic period, and the tendency noted above would underlie the sequentially-increasing divergence of transcriptome in the late embryonic period.

To further analyze types of sequences within ACRs that contribute to the recapitulative pattern, we classified ACRs into exonic and non-exonic regions. No recapitulative patterns were observed for ACRs that overlap with coding exons (Additional file 2: Figure S12), whereas non-exonic ACRs, especially those outside of promoters, exhibited recapitulative patterns (Additional file 2: Figure S12), which is essentially similar to the results shown in Fig. 3. Because sequences in exonic regions tend to be under negative selection [20, 29], it is tempting to assume that ACRs of sequences under negative selection—rather than ones overlapping coding exons—could not show the recapitulative pattern. Our additional analysis, however, did not support this. We analyzed only ACRs of sequences under negative selection using phastCons [30], which showed that chromatin accessibility of these ACRs followed a similar recapitulative pattern (Additional file 2: Figure S13). These results suggest that regulatory activity of non-coding regions, especially intronic or intergenic regions, mainly contributes to the recapitulative pattern of the chromatin accessibility.

## Discussion

Using three vertebrate species—mouse, chicken, and medaka—we found that the evolutionarily newer genomic regions (especially ones in intergenic regions and introns; Additional file 2: Figure S12) tend to become accessible at later developmental stages during mid-to-late embryogenesis. As a result, whole-embryonic chromatin accessibility appeared to follow a sequential pattern that proceeds from ancestral to derived states during mid-to-late embryogenesis (Fig. 3). This recapitulative pattern could potentially underlie gene regulatory programs pivotal for some embryonic patterns of vertebrates that are recognized as recapitulation by comparative embryologists, especially those that occur during later embryogenesis [12, 13, 16].

Although the recapitulative pattern was commonly observed in the three species analyzed, the steepness of the peak transitions along the developmental stages differed among species. This inconsistency may be explained in part by the developmental stages selected for analysis, as well as the phylogenetic distribution of analyzed categories. Detailed examinations are necessary to clarify these possibilities.

In our analysis, not all of the known regulatory elements associated with embryonic novelties could be detected. For example, our analysis based on whole embryos detected no ACRs at three representative enhancer regions possibly associated with some synapomorphies (Additional file 2: Figure S14): a mouse *Wnt5a* enhancer associated with the mammalian secondary palate [31], a chicken *Sim1* enhancer possibly associated with flight feather development [20], and a medaka *shh* enhancer possibly associated with paired appendages of the gnathostomes [32]. This could be because the numbers of cells was too small to be detected in our whole-embryo-based analysis. However, we could detect clearer examples of regulatory activities associated with overt synapomorphies (Additional file 2: Figure S14). For example, at a mouse *Satb2* enhancer region, which is responsible for the development of callosal projections [33], we detected an ACR at E14.5 when this enhancer is active in the deep layer of the neocortex [33] (Additional file 2: Figure S14). We also detected ACRs at a mouse *Fezf2* enhancer associated with the mammalian corticospinal system that drive the expression in the mouse neocortex [34] (Additional file 2: Figure S14). To fill the gap between the regulatory activities and the recapitulative development of morphological features, further detailed studies are needed, especially studies focusing on organ- or tissue-specific regulatory activities.

The evolutionary background for the observed recapitulative pattern during the diversifying later embryogenesis remains unclear. It may be that activation of newly acquired regulatory elements at earlier developmental stages leads to less adaptive phenotypes or lethality more frequently than does activation at later stages. For example, previous hypotheses [35–38] and a simulation-based study [39] predicted that earlier developmental stages are more likely to be conserved (or less evolvable [40]) because they serve as a prerequisite for later stages. Another idea worth considering is the internal selection-based hypothesis, which states that newly introduced regulatory changes are more likely to survive if they are activated during later embryogenesis due to the increasing modularity of embryos; that is, changes in one or a few modules will not affect embryonic development in its entirety [14]. However, these existing hypotheses do not fully explain the present results, as the observed recapitulative pattern is not detected before the conserved mid-embryonic period. This could be explained by developmental constraints [41] embodied at the mid-embryonic stages together with gene regulatory conservation. However, verifying this idea also requires further detailed studies.

In the present study, the higher chromatin accessibility of evolutionarily new regions at the earlier stages is compatible with the hourglass-like divergence of developmental transcriptomes [4–8]. It is possible that diversification of species-specific maternal reproductive strategies [42, 43] was facilitated by newly introduced changes in gene regulation at earlier stages. Nonetheless, the lower frequency of new regulatory activations at mid-embryonic stages than at earlier stages remains mechanistically unexplained. Further studies are needed to resolve the mechanisms underlying the recapitulative pattern from the phylotypic period and to connect evolutionary changes in gene regulation and their effects on the hourglass-like pattern of embryonic divergence in the context of modern evolutionary biology.

## Conclusions

The present study showed a recapitulative pattern in chromatin accessibility during embryogenesis from the phylotypic period onward. The observed tendency may explain, at least in part, the background for morphologically observed recapitulative embryogenesis. Although the mechanism underlying the epigenetic recapitulation remains an open question for future studies, our findings imply an evolutionary bias of developmental changes being added toward later stages in vertebrate embryogenesis.

## Methods

### Data reporting

No statistical methods were used to predetermine sample size. The investigators were not blinded to allocation during experiments or during outcome assessment.

### Animal care and use

Experimental procedures and animal care were conducted in accordance with the guidelines approved by the Animal Experiment Committee of the University of Tokyo (Animal_plan_26–3). All efforts were made to minimize animal pain and distress. Individual embryos were selected randomly from a wild-type population.

### Embryo collection

*Mus musculus*: All embryos were collected from C57BL/6 J mice (CLEA Japan) and staged according to standard morphological information on normal mouse developmental stages [44]. After removing the amniotic membranes from the staged embryos, we pooled at least two embryos from different pregnant mice to prepare each biological replicate (Additional file 1: Table S1).

*Gallus gallus*: NERA-strain fertilized chicken eggs were purchased from a local farmer in Japan (Shiroyama-keien, Kanagawa, Japan). Fertilized chicken eggs were incubated at 38 °C in a humidified incubator and morphologically staged according to the Hamburger–Hamilton system [45]. After the amniotic membranes had been removed from staged individual embryos, we pooled at least two embryos to prepare biological replicates of each developmental stage (Additional file 1: Table S1).

*Oryzias latipes*: Mature adults of the d-rR strain were maintained under standard conditions (10:14-h dark:light cycle; 26–28 °C) and mated to obtain fertilized eggs. Fertilized eggs were incubated at 24–26 °C, and individual embryos were morphologically staged, as described by Iwamatsu [46]. Biological replicates comprised pooled embryos from different pairs of parents (Additional file 1: Table S1).

### Preparation and sequencing of the ATAC-seq library

For each biological replicate, ATAC-seq was performed as previously described [47, 48], with some modifications. In brief, embryos were minced as required by using a razor blade, placed in homogenization buffer (25 mM D-sucrose, 20 mM tricine [pH 7.8], 15 mM NaCl, 60 mM KCl, 2 mM MgCl_2_, 0.5 mM spermidine, and cOmplete Protease Inhibitor Cocktail Tablet [Roche]), and homogenized in an ice-cold Dounce tissue grinder with a loose-fitting pestle, according to the methods of Yue et al. [49]. For further dissociation, each homogenized sample was forced through a 21-gauge needle by using a syringe and then filtered through a 100-μm Filcon filter (As One Corporation). Cells were harvested by centrifugation at 500×*g* for 5 min at 4 °C and then resuspended in 500 μL of cold sucrose buffer (250 mM D-sucrose, 10 mM Tris HCl [pH 7.5], 1 mM MgCl_2_, and cOmplete Protease Inhibitor Cocktail Tablet). In total, 500,000 cells or 50,000 cells was centrifuged at 500×*g* for 5 min at 4 °C for each sample, depending on the developmental stage (Additional file 1: Table S1). Cells were resuspended in 50 μL of cold lysis buffer (10 mM Tris HCl [pH 7.4], 10 mM NaCl, 3 mM MgCl_2_, 0.1% v/v Igepal CA-630 [Sigma–Aldrich]) and incubated on ice for 5 min. After centrifugation of the cells at 500×*g* for 10 min at 4 °C, the supernatant was discarded. Tagmentation reactions were performed at 37 °C for 30 min by using a Nextera Sample Preparation Kit (Illumina). Tagmentated DNA was purified by using a DNA concentrator kit (Zymo Research) and size-selected (< 500 bp) by using AMPure XP beads (Beckman Coulter). Next, to enrich for small DNA fragments, two sequential PCR amplifications were performed, as described previously [47]. The amplified DNA was purified by using a DNA concentrator kit (Zymo Research), and the quality of the purified library was assessed by using a 2100 Bioanalyzer (Agilent Technologies) and a High Sensitivity DNA analysis kit (Agilent Technologies) to confirm a periodic pattern in the size of the amplified DNA. After size selection (100–300 bp) by using AMPure XP beads, the libraries were sequenced as paired-end 50-bp reads by using the HiSeq 1500 platform (Illumina) according to the manufacturer’s instructions.

### Alignment of ATAC-seq reads

Adaptor trimming and quality filtering of raw paired-end reads were performed by using Trimmomatic (version 0.36) [50] with the following parameters: ILLUMINACLIP: adaptor.fa:2:30:10, LEADING: 20, TRAILING: 20, SLIDINGWINDOW: 4:15, MINLEN: 36. The filtered reads were aligned to species-specific hard-masked reference genomes (GRCm38 for *Mus musculus* [51], Gallus_gallus-5.0 for *Gallus gallus* [52], HdrR for *Oryzias latipes* [53]) by using bowtie2 (version 2.2.6) [54] with the following parameters: -k 4 -X 2000 --sensitive. These genomes were downloaded from the Ensembl database (release 89) [55]. Picard (version 2.9.0 [56]) was used to remove duplicate reads from among properly paired aligned reads, which had been filtered by using SAMtools (version 1.4) [57]. For the analysis with uniquely hit ATAC-seq reads, we extracted only uniquely aligned reads after duplicate reads had been removed. Finally, for each sample, we randomly selected 20 million aligned reads to create a read-depth-controlled dataset for further analysis. For analyses without reads aligned to the mitochondrial genome and with uniquely hit ATAC-seq reads, we randomly selected 18 million aligned reads for further analysis. The Integrative Genomics Viewer [58] was used to visualize enrichment of ATAC-seq reads at a window size of 20 bp.

### Identification of ACRs

To identify ACRs that were consistently present across three biological replicates, ATAC-seq peaks were called according to the method described by Daugherty et al. [59] by using MACS (version 2.1.1) [60]. The start position of every aligned ATAC-seq read was first corrected to account for the 9-bp insert between the adaptors, which was introduced by Tn5 transposase [22], and then the single 5′-most base of each read was retained to obtain single-base resolution. These data for all biological replicates were pooled into a single dataset. For the pooled data and those for each replicate, peak calling was performed by using MACS at a moderate threshold (−-nomodel --extsize 50 --shift − 25 -p 0.1 --keep-dup all). Of the peaks in the pooled data, we retained those that had at least 50% overlap with peaks in all replicates. The ATAC-seq signal intensity of each ACR was calculated by the number of 5′ ends of reads within it. To evaluate the reproducibility of whole-embryo chromatin accessibility between biological replicates, we calculated Pearson correlation coefficients by using the ATAC-seq signal intensities (log10-RPM; reads per million mapped reads) of ACRs at different developmental stages. To examine the percentage of known enhancers overlapping with the identified ACRs, the mouse ACRs at any of the developmental stages were compared with the mouse VISTA enhancers, which were downloaded from the VISTA Enhancer Database [23]. Mm9 coordinates of VISTA enhancers were converted to GRCm38 using liftOver [61].

### Analysis of genomic distribution of ACRs

To examine the genomic distribution of ACRs in each species (Additional file 2: Figure S2a), all ACRs at all developmental stages were consolidated into a single list. In this list, overlapping ACRs were combined into a single ACR. These ACRs were classified into five groups: (1) ACRs in promoters were defined as those overlapping with regions between 2 kb upstream and 1 kb downstream of all TSSs of protein-coding genes, which are retrieved from the Ensembl database [55]; (2) ACRs in exons were defined as those overlapping with exons of protein-coding genes but not overlapping with promoters; (3) ACRs in introns were defined as those in gene bodies of protein-coding genes but not overlapping with either exons or promoters; (4) proximal ACRs were defined as those overlapping with regions between 5 kb upstream and 1 kb downstream of all TSSs but not overlapping with either promoters or gene bodies; (5) distal ACRs were defined as those not in groups 1–4. To test whether the ACRs were statistically enriched at promoters, we performed a two-sided Fisher’s exact test. As control data in this test, we randomly chose regions with the same lengths of individual ACRs among the reference genome, not allowing chosen regions to overlap each other. For the analysis illustrated in Additional file 2: Figure S2b, the ACRs in promoters were further grouped into five groups according to their distance from annotated TSSs.

### Quantification of FRiP scores

To quantitatively evaluate the signal-to-background ratio of whole-embryo ATAC-seq data in a genome-wide manner, we calculated the fraction of all aligned reads in the ACRs (FRiP scores) [62]. The FRiP score is used as a quality metric of the signal-to-background ratio for ATAC-seq data in the ENCODE consortium [63]. For each developmental stage, we first identified ACRs with properly aligned non-duplicate reads and then quantified the FRiP score. According to the quality standard defined by the ENCODE consortium, the ATAC-seq data with FRiP scores > 0.2 are acceptable.

### Whole-genome pairwise alignment

To estimate the evolutionary age of each genomic region, we generated whole-genome pairwise alignments against different sets of animal genomes (Additional file 1: Table S6) [7, 19, 51–53, 64–87]. To avoid a computational barrier, unplaced scaffolds of mouse, chicken, and medaka were removed from the hard-masked genome sequences and only chromosomal sequences were used. The reference genomes were split into 30-Mb sequences, and the query genomes were split into 10-Mb sequences with 100 kb of overlap. Pairwise genome alignments of them were performed using LASTZ (version 1.04.00) [88] with the following parameters: --seed = 12of19 --transition --step = 1 --strand = both --nochain --gapped --scores = HOXD70 --gap = 400,30 --xdrop = 910 --ydrop = 9400 --hspthresh = 3000 --inner = 2200 --gappedthresh = 3000 --entropy --masking = 0. The obtained local alignments were then combined into the chained alignments using axtChain [89] with the following parameters: -minScore = 3000 -linearGap = medium or -minScore = 5000 -linearGap = loose, depending on the phylogenetic distance between the two aligned species (Additional file 1: Table S6). After removing chains derived from repeats using chainAntiRepeat [89], we converted the chained alignments into netted alignments using chainSort, chainPreNet, and chainNet [89] and then added syntenic information to them using netSyntenic [89]. These alignments were used for estimating evolutionary ages of ACRs, as described in the following section.

For identification of genomic regions under strong negative selection, we used PHAST [30, 90, 91]. First, we constructed multiple alignments from the pairwise alignments. Among aligned sequences, we extracted reciprocal-best alignments to identify the best-conserved regions. Using extracted pairwise alignments, we created multiple alignments with Multiz (version 11.2) [92] and ROAST (version 3 [93]) for each of the reference species. Next, with the multiple alignments of each species, we used phyloFit [90] with the tree topology of genomes shown in Fig. 1a to build a neutral model based on four-fold degenerative sites of protein-coding regions. Using this neutral model and multiple alignments as the input, to identify the regions under strong negative selection we used phastCons [30] with the following parameters: --target-coverage 0.3 --expected-length 12 --rho 0.3 --most-conserved. To validate that our analysis was sufficiently sensitive to identify regions under negative selection, we compared these regions with coding exons for each of the reference species. These identified regions overlapped with 83.7, 82.0, and 81.3% of protein-coding exons in mouse, chicken, and medaka, respectively, indicating that we were able to sensitively detect regions under negative selection. In the analysis for Additional file 2: Figure S13, the ATAC-seq signal intensities of the conserved regions under negative selection within ACRs were summed for each evolutionary category, and their ratios relative to the total signal intensities of all the ACRs in all the evolutionary categories were calculated (relative ATAC-seq signals). It should also be noted that none of the species-specific ACRs are categorized under negative selection, because the regions under negative selection detected by phastCons are required to be aligned against one or more other species genomes.

### Estimation of evolutionary ages of ACRs

To estimate evolutionary ages of the ACRs, we used the whole genome alignment dataset. For robust estimation of evolutionary ages of ACRs, we performed four methods (methods I–IV; Additional file 2: Figure S4a, b). In methods I and II, for each ACR we first determined the species that share sequences similar to those of the ACR, and the evolutionary age was then defined as the time span from the most recent common ancestor of all the analyzed species sharing a similar sequence of the ACR. ACRs consisting of multiple regions of different evolutionary ages (40% in mouse, 34% in chicken, and 46% in medaka) were subdivided into separate ACRs. In method I, ACRs that were estimated to be lost secondarily in any of the aligned species were excluded in order to focus only on ACRs shared by all the analyzed species of a certain monophyletic group and by no additional outgroup species. On the other hand, method II did not exclude ACRs that had been lost secondarily. In contrast to methods I and II, no ACRs were subdivided in methods III and IV. For each ACR, we determined the species that share the sequences similar to at least one-third of the regions of the ACR, and then the evolutionary age was defined as the time span from the most recent common ancestor of all these species. As for secondarily lost ACRs, method III excluded them, whereas method IV did not.

### ATAC-seq data from mouse pre-implantation embryos

In the analysis including developmental stages before E7.5 in mouse (Additional file 2: Figure S11), we used previously published ATAC-seq datasets (GEO accession: GSM1933921 and GSM1933922 for early two-cell stage; GSM1933924 and GSM1933925 for two-cell stage; GSM1625847 and GSM1933927 for four-cell stage; GSM1933928 and GSM1933929 for eight-cell stage) [28]. As we did for the ATAC-seq data generated in this study, the ATAC-seq reads were filtered and then aligned to the mouse reference genome. In these ATAC-seq data of mouse pre-implantation embryos, the average mitochondrial fraction was as much as 43.2% of properly aligned non-duplicated reads, which is considerably higher than those of later staged mouse embryos (~ 8.7%; see Additional file 3: Text S2.4). Thus, for the analysis illustrated in Additional file 2: Figure S11, ATAC-seq reads aligned to the mitochondrial genome were removed. In addition, we did not perform read-depth normalization, because the numbers of the aligned reads in the ATAC-seq data from the pre-implantation embryos did not reach the threshold for read-depth normalization (i.e., 18 million reads).

### Cross-species transcriptome comparison

Previously published [7, 8, 94] RNA-seq datasets were used to calculate early to late whole-embryo gene expression profiles (DDBJ accession DRA003460 for mouse [*M. musculus*], chicken [*G. gallus*], softshell turtle [*Pelodiscus sinensis*], western clawed frog [*Xenopus tropicalis*], and zebrafish [*Danio rerio*]; DRA005309 for medaka [*O. latipes*]). In brief, RNA-seq reads were aligned to each reference genome [7, 51–53, 68, 87] by using Tophat2 (version 2.0.14) [95] with the following parameters: -g 1 -N 3 --read-edit-dist 3. For paired-end RNA-seq data of medaka embryos, among all aligned reads only properly paired aligned reads with a primary alignment were then filtered by using SAMtools (version 1.4) [57]. To obtain expression levels of genes on the basis of these aligned datasets, FPKM (fragments per kilobase of transcripts per million fragments mapped) values were calculated by using Cufflinks (version 2.2.1) [96] and a gene set retrieved from the Ensembl database [55]. For interspecific comparisons of orthologous gene expression levels, we used 1:1 orthologue information between each pair of species that was obtained from the Ensembl Compara Database through BioMart [97]. To evaluate transcriptome similarity between samples, a Spearman correlation coefficient was calculated by using the expression values of orthologous genes, as described by Wang et al. [7]. As reported previously [8, 98], phylogenetic relationships between the species being compared were considered in the transcriptome-based identification of vertebrate-conserved stages. For stage combinations among the six different vertebrate embryos (mouse, chicken, softshell turtle, western clawed frog, zebrafish, and medaka), we extracted pairs of species that reflected the phylogenetic scale of interest (i.e., vertebrates) and took their average value as expDist. To estimate the most conserved stages, we first identified stage combinations with the most similar expression profiles of 1:1 orthologues (lowest 1% expDists) from all combinations. We then visualized the contribution of each stage in medaka embryogenesis to this top 1% of similarly staged embryo combinations as P_top_ (percentage of stage included in the top similar 1% of stage–embryo combinations). This calculation process was performed 100 times with randomly selected biological replicate data to derive statistically robust conclusions. When 1:1 orthologues were used in this analysis, genes that lacked 1:1 orthologue counterparts in any of the species (e.g., because of gene loss in any of the species being compared) were ignored, as previously described [8]. The other dataset for calculating expDists was the orthologue-group-based gene expression profiles, in which expression levels of in-paralogs defined by orthoMCL [99] were summed and further compared between species to calculate expression distances.

### Estimation of evolutionary ages of protein-coding genes

We used peptide sequences from mouse, chicken, and medaka to estimate the evolutionary ages of protein-coding genes. After removing entries shorter than 30 amino acids, we selected the longest peptide sequence for each gene and used it in tblastn searches (version 2.7.1) with a threshold E-value < 10^− 10^ to identify regions with similar sequences in the other species’ genomes. Using this information, we estimated the evolutionary age of each protein-coding gene based on the most recent common ancestors of all these species. As with the analysis of ACRs, we prepared two different datasets to ensure that the methods to estimate the evolutionary ages did not influence our conclusions. One dataset includes only the protein-coding genes that are present in all the analyzed species of a certain monophyletic group, but not found in any outgroup species (Fig. 4 and Additional file 2: Figure S7). In the other dataset, all protein-coding genes were included. The results using both datasets were similar (data of second analysis not shown). At each developmental stage, the total expression level of genes with the same evolutionary age were calculated by using the previously determined FPKM-normalized whole-embryo gene expression profiles of mouse, chicken, and medaka [8, 94].

### Software

In addition to software specified elsewhere in the Methods, we used bedtools (version 2.27.1) [100] and ggplot2 [101]. Phylogenetic relationships and split times were adopted from the TimeTree database [102] (Fig. 1a and Additional file 2: Figures S1, 4, 5).

### Statistical analysis

We used R (ver. 3.4.4) [103] to perform all statistical analyses. An alpha level of 0.05 was adopted for statistical significance throughout the analyses. For the statistical analyses illustrated in Figs. 3 and 4 and Additional file 2: Figures S6–13, the Kruskal–Wallis rank sum test was used. In case of multiple comparisons, the Benjamini–Hochberg procedure [104] was used to control the false discovery rates at 0.05. For the analyses shown in Additional file 2: Figure S1a–e and S2, correlation coefficients were regarded as valid only when the comparison was confirmed to have a significant correlation by a test of no correlation. For statistical analyses illustrated in Additional file 2: Figure S1f, the Friedman rank sum test was used. Details of the statistical analyses used in this study are provided in Additional file 1.

## Supplementary information

**Additional file 1 Table S1.** Information on whole-embryo ATAC-seq samples. **Table S2.** Numbers of ACRs at each developmental stage. **Table S3.** FRiP score of whole-embryo ATAC-seq data. **Table S4.** The numbers of ACRs at promoters and the two-sided Fisher’s exact test. **Table S5.** Biological reproducibility of whole-embryo ATAC-seq signal intensities. **Table S6.** Information on whole-genome pairwise alignment data. **Tables S7 and S8.** Statistical information in Figs. 3 and 4. **Table S9.** Detailed information on representative enhancers from the VISTA Enhancer Database. **Tables S10–S22.** Statistical information in Figures S7–S13.

**Additional file 2 Figure S1.** Identification of conserved mid-embryonic stages during medaka embryogenesis by transcriptome similarity. **Figure S2.** Genomic distribution of mouse, chicken, and medaka ACRs with respect to genome annotations. **Figure S3.** Enhancers that drive wider expression tend to have higher ATAC-seq signals. **Figure S4.** Four methods for estimating evolutionary ages of ACRs. **Figure S5.** Categorization of evolutionary ages of ACRs and expressed protein-coding genes did not differ among different methods. **Figure S6.** The recapitulative pattern was also observed for relative sequence length of evolutionarily categorized ACRs within the genome. **Figure S7.** Developmental gene expression levels did not show a recapitulative pattern in the analysis including genes lost secondarily, related to Fig. 4. **Figure S8.** The recapitulative pattern of whole-embryo chromatin accessibility was consistent between methods I, II, III, and IV, related to Fig. 3. **Figure S9.** Essentially the same recapitulative pattern was observed in the analysis using different sets of species. **Figure S10.** Essentially the same recapitulative pattern was observed for the analyses with different criteria in filtering ATAC-seq reads. **Figure S11.** Chromatin accessibility of mouse early stages did not show the recapitulative pattern. **Figure S12.** Exonic ACRs did not follow a recapitulative pattern. **Figure S13.** Similar recapitulative pattern observed for the chromatin accessibility under strong negative selection. **Figure S14.** No ACR could be detected at three of five representative regulatory regions associated with taxon-specific features.

**Additional file 3 Text S1.** The phylotypic period in medaka embryogenesis. **Text S2.** Testing the recapitulative pattern with the different datasets.

## Data Availability

All ATAC-seq data generated during the current study are available in the DNA Data Bank of Japan, [https://trace.ddbj.nig.ac.jp/DRASearch/submission?acc=DRA006971].
