# Supplementary material for: Recapitulation-like developmental transitions of chromatin accessibility in vertebrates
Source: Zoological Lett. 2019 Nov 14;5:33. doi: 10.1186/s40851-019-0148-9 (PMC6857340; doi:10.1186/s40851-019-0148-9)
Supplement: Supplementary file 1 — Additional file 1 Table S1. Information on whole-embryo ATAC-seq samples. Table S2. Numbers of ACRs at each developmental stage. Table S3. FRiP score of whole-embryo ATAC-seq data. Table S4. The numbers of ACRs at promoters and the two-sided Fisher’s exact test. Table S5. Biological reproducibility of whole-embryo ATAC-seq signal intensities. Table S6. Information on whole-genome pairwise alignment data. Tables S7 and S8. Statistical information in Figs. 3 and 4. Table S9. Detailed information on representative enhancers from the VISTA Enhancer Database. Tables S10–S22. Statistical information in Figures S7–S13. [file 40851_2019_148_MOESM1_ESM.pdf]

Additional file 1: Supplementary Tables

Table S1. Information on whole-embryo ATAC-seq samples.

Species	Developmental stage	Biological replicate number	Number of embryos pooled	Number of cells for ATAC-seq	Filtered reads
Mouse	E7.5	1	16	50,000	163,806,138
	E7.5	2	18	50,000	150,590,012
	E7.5	3	15	50,000	123,878,902
	E8.5	1	15	50,000	146,637,870
	E8.5	2	6	50,000	160,013,422
	E8.5	3	14	50,000	139,587,780
	E9.5	1	6	500,000	133,768,752
	E9.5	2	15	500,000	152,951,300
	E9.5	3	6	500,000	138,861,218
	E10.5	1	6	500,000	152,238,300
	E10.5	2	8	500,000	149,835,156
	E10.5	3	8	500,000	141,327,110
	E12.5	1	2	500,000	143,046,480
	E12.5	2	2	500,000	139,967,892
	E12.5	3	2	500,000	172,080,896
	E14.5	1	2	500,000	148,176,792
	E14.5	2	2	500,000	139,462,932
	E14.5	3	2	500,000	139,744,522
	E16.5	1	2	500,000	137,658,622
	E16.5	2	2	500,000	161,752,784
	E16.5	3	2	500,000	121,824,564
	E18.5	1	2	500,000	147,274,838
	E18.5	2	2	500,000	171,704,282
	E18.5	3	2	500,000	168,567,462
Chicken	HH6	1	5	500,000	150,293,014
	HH6	2	4	500,000	160,558,314
	HH6	3	4	500,000	144,524,764
	HH11	1	5	500,000	133,312,472
	HH11	2	5	500,000	129,538,096
	HH11	3	4	500,000	135,804,384
	HH16	1	7	500,000	136,976,744
	HH16	2	7	500,000	138,557,562
	HH16	3	7	500,000	129,294,694
	HH19	1	4	500,000	131,750,406
	HH19	2	4	500,000	152,140,888
	HH19	3	4	500,000	158,072,072
	HH24	1	2	500,000	139,759,998
	HH24	2	2	500,000	137,259,058
	HH24	3	2	500,000	152,682,324
	HH28	1	2	500,000	124,272,006
	HH28	2	2	500,000	116,448,198
	HH28	3	2	500,000	122,776,106
	HH32	1	2	500,000	142,818,500
	HH32	2	2	500,000	145,458,928
	HH32	3	2	500,000	157,787,170
	HH38	1	2	500,000	156,110,422
	HH38	2	2	500,000	142,826,992
	HH38	3	2	500,000	148,502,578
Medaka	Stage 15	1	73	50,000	146,821,406

	Stage 15	2	74	50,000	122,253,936
	Stage 15	3	73	50,000	111,469,822
	Stage 21	1	40	50,000	315,598,798
	Stage 21	2	30	50,000	218,284,158
	Stage 21	3	37	50,000	303,105,284
	Stage 24	1	66	500,000	185,387,768
	Stage 24	2	40	500,000	214,447,928
	Stage 24	3	40	500,000	201,949,114
	Stage 28	1	30	500,000	136,739,522
	Stage 28	2	30	500,000	134,723,176
	Stage 28	3	37	500,000	143,599,588
	Stage 32	1	20	500,000	149,691,470
	Stage 32	2	24	500,000	120,335,888
	Stage 32	3	19	500,000	153,597,756
	Stage 36	1	17	500,000	123,633,866
	Stage 36	2	24	500,000	86,787,606
	Stage 36	3	17	500,000	169,536,716
	Stage 40	1	9	500,000	116,558,148
	Stage 40	2	3	500,000	197,537,256
	Stage 40	3	6	500,000	138,102,580

Table S2. Numbers of ACRs at each developmental stage.

Species	Developmental stage	Number of ACRs
Mouse	E7.5	159,511
	E8.5	220,908
	E9.5	226,497
	E10.5	232,539
	E12.5	258,350
	E14.5	232,001
	E16.5	234,434
	E18.5	263,158
Chicken	HH6	292,196
	HH11	297,167
	HH16	302,889
	HH19	310,539
	HH24	329,279
	HH28	342,874
	HH32	354,700
	HH38	304,751
Medaka	Stage 15	191,151
	Stage 21	200,464
	Stage 24	214,908
	Stage 28	199,302
	Stage 32	213,963
	Stage 36	211,107
	Stage 40	187,170

Table S3. FRiP score of whole-embryo ATAC-seq data.

Species	Developmental stage	FRiP score*
Mouse	E7.5	0.264
	E8.5	0.393
	E9.5	0.366
	E10.5	0.405
	E12.5	0.386
	E14.5	0.313
	E16.5	0.264
	E18.5	0.201
Chicken	HH6	0.274
	HH11	0.354
	HH16	0.530
	HH19	0.539
	HH24	0.470
	HH28	0.418
	HH32	0.412
	HH38	0.301
Medaka	Stage 15	0.279
	Stage 21	0.274
	Stage 24	0.374
	Stage 28	0.430
	Stage 32	0.454
	Stage 36	0.472
	Stage 40	0.383

*The FRiP score is a genome-wide, quality metric of the signal-to-background ratio, and it is defined as a fraction of all aligned reads in the called peak regions [62]. According to the quality standard defined by the ENCODE consortium [63], the ATAC-seq data with >0.2 FRiP scores are acceptable.

Table S4. The numbers of ACRs at promoters and the two-sided Fisher's exact test.

	ACRs		Random regions		
Species	in promoters	outside of promoters	in promoters	outside of promoters	Two-sided Fisher's exact test (*P*)
Mouse	81,245	656,850	37,253	700,842	$< 2.2 \times 10^{-16}$
Chicken	45,480	753,032	35,867	762,645	$< 2.2 \times 10^{-16}$
Medaka	53,411	393,986	32,273	415,124	$< 2.2 \times 10^{-16}$

Table S5. Biological reproducibility of whole-embryo ATAC-seq signal intensities.

Species	Stage	Pair compared	Pearson correlation coefficient (r)	*T* of test for no correlation	*P* value
Mouse	E7.5	Rep. 1 vs Rep. 2	0.859	668.907	$< 2.2 \times 10^{-16}$
		Rep. 1 vs Rep. 3	0.855	658.630	$< 2.2 \times 10^{-16}$
		Rep. 2 vs Rep. 3	0.905	847.750	$< 2.2 \times 10^{-16}$
	E8.5	Rep. 1 vs Rep. 2	0.903	989.000	$< 2.2 \times 10^{-16}$
		Rep. 1 vs Rep. 3	0.908	1018.322	$< 2.2 \times 10^{-16}$
		Rep. 2 vs Rep. 3	0.891	924.761	$< 2.2 \times 10^{-16}$
	E9.5	Rep. 1 vs Rep. 2	0.891	932.351	$< 2.2 \times 10^{-16}$
		Rep. 1 vs Rep. 3	0.886	907.021	$< 2.2 \times 10^{-16}$
		Rep. 2 vs Rep. 3	0.901	986.235	$< 2.2 \times 10^{-16}$
	E10.5	Rep. 1 vs Rep. 2	0.909	1052.135	$< 2.2 \times 10^{-16}$
		Rep. 1 vs Rep. 3	0.898	985.457	$< 2.2 \times 10^{-16}$
		Rep. 2 vs Rep. 3	0.911	1067.454	$< 2.2 \times 10^{-16}$
	E12.5	Rep. 1 vs Rep. 2	0.897	1028.852	$< 2.2 \times 10^{-16}$
		Rep. 1 vs Rep. 3	0.901	1053.518	$< 2.2 \times 10^{-16}$
		Rep. 2 vs Rep. 3	0.891	994.911	$< 2.2 \times 10^{-16}$
	E14.5	Rep. 1 vs Rep. 2	0.900	992.272	$< 2.2 \times 10^{-16}$
		Rep. 1 vs Rep. 3	0.903	1009.557	$< 2.2 \times 10^{-16}$
		Rep. 2 vs Rep. 3	0.899	986.149	$< 2.2 \times 10^{-16}$
	E16.5	Rep. 1 vs Rep. 2	0.895	969.709	$< 2.2 \times 10^{-16}$
		Rep. 1 vs Rep. 3	0.892	953.606	$< 2.2 \times 10^{-16}$
		Rep. 2 vs Rep. 3	0.898	988.806	$< 2.2 \times 10^{-16}$
	E18.5	Rep. 1 vs Rep. 2	0.846	812.949	$< 2.2 \times 10^{-16}$
		Rep. 1 vs Rep. 3	0.849	823.157	$< 2.2 \times 10^{-16}$
		Rep. 2 vs Rep. 3	0.850	829.223	$< 2.2 \times 10^{-16}$
Chicken	HH6	Rep. 1 vs Rep. 2	0.884	1023.111	$< 2.2 \times 10^{-16}$
		Rep. 1 vs Rep. 3	0.881	1005.550	$< 2.2 \times 10^{-16}$
		Rep. 2 vs Rep. 3	0.883	1018.269	$< 2.2 \times 10^{-16}$
	HH11	Rep. 1 vs Rep. 2	0.912	1208.464	$< 2.2 \times 10^{-16}$
		Rep. 1 vs Rep. 3	0.899	1120.904	$< 2.2 \times 10^{-16}$
		Rep. 2 vs Rep. 3	0.899	1118.204	$< 2.2 \times 10^{-16}$
	HH16	Rep. 1 vs Rep. 2	0.923	1320.017	$< 2.2 \times 10^{-16}$
		Rep. 1 vs Rep. 3	0.924	1329.981	$< 2.2 \times 10^{-16}$
		Rep. 2 vs Rep. 3	0.924	1325.296	$< 2.2 \times 10^{-16}$
	HH19	Rep. 1 vs Rep. 2	0.927	1379.717	$< 2.2 \times 10^{-16}$
		Rep. 1 vs Rep. 3	0.926	1368.087	$< 2.2 \times 10^{-16}$
		Rep. 2 vs Rep. 3	0.930	1410.105	$< 2.2 \times 10^{-16}$
	HH24	Rep. 1 vs Rep. 2	0.912	1274.164	$< 2.2 \times 10^{-16}$
		Rep. 1 vs Rep. 3	0.901	1191.306	$< 2.2 \times 10^{-16}$
		Rep. 2 vs Rep. 3	0.911	1267.540	$< 2.2 \times 10^{-16}$
	HH28	Rep. 1 vs Rep. 2	0.906	1251.740	$< 2.2 \times 10^{-16}$
		Rep. 1 vs Rep. 3	0.908	1270.402	$< 2.2 \times 10^{-16}$
		Rep. 2 vs Rep. 3	0.906	1253.564	$< 2.2 \times 10^{-16}$
	HH32	Rep. 1 vs Rep. 2	0.910	1308.219	$< 2.2 \times 10^{-16}$
		Rep. 1 vs Rep. 3	0.908	1288.459	$< 2.2 \times 10^{-16}$
		Rep. 2 vs Rep. 3	0.906	1273.837	$< 2.2 \times 10^{-16}$
	HH38	Rep. 1 vs Rep. 2	0.897	1121.290	$< 2.2 \times 10^{-16}$
		Rep. 1 vs Rep. 3	0.899	1134.043	$< 2.2 \times 10^{-16}$
		Rep. 2 vs Rep. 3	0.903	1158.655	$< 2.2 \times 10^{-16}$
Medaka	Stage 15	Rep. 1 vs Rep. 2	0.906	933.672	$< 2.2 \times 10^{-16}$
		Rep. 1 vs Rep. 3	0.907	940.531	$< 2.2 \times 10^{-16}$
		Rep. 2 vs Rep. 3	0.906	935.824	$< 2.2 \times 10^{-16}$
	Stage 21	Rep. 1 vs Rep. 2	0.911	991.763	$< 2.2 \times 10^{-16}$
		Rep. 1 vs Rep. 3	0.911	987.852	$< 2.2 \times 10^{-16}$
		Rep. 2 vs Rep. 3	0.909	979.181	$< 2.2 \times 10^{-16}$
	Stage 24	Rep. 1 vs Rep. 2	0.872	825.500	$< 2.2 \times 10^{-16}$
		Rep. 1 vs Rep. 3	0.874	834.035	$< 2.2 \times 10^{-16}$
		Rep. 2 vs Rep. 3	0.931	1179.750	$< 2.2 \times 10^{-16}$
	Stage 28	Rep. 1 vs Rep. 2	0.929	1122.782	$< 2.2 \times 10^{-16}$
		Rep. 1 vs Rep. 3	0.928	1115.674	$< 2.2 \times 10^{-16}$

	Rep. 2 vs Rep. 3	0.924	1081.386	$< 2.2 \times 10^{-16}$
Stage 32	Rep. 1 vs Rep. 2	0.928	1156.388	$< 2.2 \times 10^{-16}$
	Rep. 1 vs Rep. 3	0.924	1117.229	$< 2.2 \times 10^{-16}$
	Rep. 2 vs Rep. 3	0.915	1051.375	$< 2.2 \times 10^{-16}$
Stage 36	Rep. 1 vs Rep. 2	0.929	1157.204	$< 2.2 \times 10^{-16}$
	Rep. 1 vs Rep. 3	0.935	1206.773	$< 2.2 \times 10^{-16}$
	Rep. 2 vs Rep. 3	0.931	1171.902	$< 2.2 \times 10^{-16}$
Stage 40	Rep. 1 vs Rep. 2	0.898	883.883	$< 2.2 \times 10^{-16}$
	Rep. 1 vs Rep. 3	0.901	899.696	$< 2.2 \times 10^{-16}$
	Rep. 2 vs Rep. 3	0.917	993.256	$< 2.2 \times 10^{-16}$

Table S6. Information on whole-genome pairwise alignment data.

Reference species	Reference genome version	Query species	Query genome version	Parameter for axtChain
Mouse (*Mus musculus*)	GRCm38	Rat (*Rattus norvegicus*)	Rnor_6.0	-minScore=3000 -linearGap=medium
Mouse (*Mus musculus*)	GRCm38	Rabbit (*Oryctolagus cuniculus*)	OryCun2.0	-minScore=3000 -linearGap=medium
Mouse (*Mus musculus*)	GRCm38	Human (*Homo sapiens*)	GRCh38	-minScore=3000 -linearGap=medium
Mouse (*Mus musculus*)	GRCm38	Cow (*Bos taurus*)	UMD3.1	-minScore=5000 -linearGap=loose
Mouse (*Mus musculus*)	GRCm38	Opossum (*Monodelphis domestica*)	monDom5	-minScore=5000 -linearGap=loose
Mouse (*Mus musculus*)	GRCm38	Chicken (*Gallus gallus*)	Gallus_gallus-5.0	-minScore=5000 -linearGap=loose
Mouse (*Mus musculus*)	GRCm38	Western clawed frog (*Xenopus tropicalis*)	JGI_4.2	-minScore=5000 -linearGap=loose
Mouse (*Mus musculus*)	GRCm38	Coelacanth (*Latimeria chalumnae*)	LatCha1	-minScore=5000 -linearGap=loose
Mouse (*Mus musculus*)	GRCm38	Spotted gar (*Lepisosteus oculatus*)	LepOcu1	-minScore=5000 -linearGap=loose
Mouse (*Mus musculus*)	GRCm38	Elephant shark (*Callorhinchus milii*)	calMil1	-minScore=5000 -linearGap=loose
Mouse (*Mus musculus*)	GRCm38	Lamprey (*Petromyzon marinus*)	Pmarinus_7.0	-minScore=5000 -linearGap=loose
Mouse (*Mus musculus*)	GRCm38	Vase tunicate (*Ciona intestinalis*)	KH	-minScore=5000 -linearGap=loose
Mouse (*Mus musculus*)	GRCm38	Amphioxus (*Branchiostoma floridae*)	v1.0	-minScore=5000 -linearGap=loose
Mouse (*Mus musculus*)	GRCm38	Roundworm (*Caenorhabditis elegans*)	WBcel235	-minScore=5000 -linearGap=loose
Mouse (*Mus musculus*)	GRCm38	Starlet sea anemone (*Nematostella vectensis*)	ASM20922v1	-minScore=5000 -linearGap=loose
Mouse (*Mus musculus*)	GRCm38	Choanoflagellate (*Salpingoeca rosetta*)	Proterospongia_ sp_ATCC50818	-minScore=5000 -linearGap=loose
Chicken (*Gallus gallus*)	Gallus_gallus-5.0	Turkey (*Meleagris gallopavo*)	UMD2	-minScore=3000 -linearGap=medium
Chicken (*Gallus gallus*)	Gallus_gallus-5.0	Zebra finch (*Taeniopygia guttata*)	taeGut3.2.4	-minScore=3000 -linearGap=medium
Chicken (*Gallus gallus*)	Gallus_gallus-5.0	American alligator (*Alligator mississippiensis*)	ASM28112v4	-minScore=5000 -linearGap=loose
Chicken (*Gallus gallus*)	Gallus_gallus-5.0	Softshell turtle (*Pelodiscus sinensis*)	PelSin_1.0	-minScore=5000 -linearGap=loose
Chicken (*Gallus gallus*)	Gallus_gallus-5.0	Anole lizard (*Anolis carolinensis*)	AnoCar2.0	-minScore=5000 -linearGap=loose
Chicken (*Gallus gallus*)	Gallus_gallus-5.0	Mouse (*Mus musculus*)	GRCm38	-minScore=5000 -linearGap=loose
Chicken (*Gallus gallus*)	Gallus_gallus-5.0	Western clawed frog (*Xenopus tropicalis*)	JGI_4.2	-minScore=5000 -linearGap=loose
Chicken	Gallus_gallus-5.0	Coelacanth	LatCha1	-minScore=5000

(*Gallus gallus*)		(*Latimeria chalumnae*)		-linearGap=loose
Chicken (*Gallus gallus*)	Gallus_gallus-5.0	Spotted gar (*Lepisosteus oculatus*)	LepOcu1	-minScore=5000 -linearGap=loose
Chicken (*Gallus gallus*)	Gallus_gallus-5.0	Elephant shark (*Callorhinchus milii*)	calMil1	-minScore=5000 -linearGap=loose
Chicken (*Gallus gallus*)	Gallus_gallus-5.0	Lamprey (*Petromyzon marinus*)	Pmarinus_7.0	-minScore=5000 -linearGap=loose
Chicken (*Gallus gallus*)	Gallus_gallus-5.0	Vase tunicate (*Ciona intestinalis*)	KH	-minScore=5000 -linearGap=loose
Chicken (*Gallus gallus*)	Gallus_gallus-5.0	Amphioxus (*Branchiostoma floridae*)	v1.0	-minScore=5000 -linearGap=loose
Chicken (*Gallus gallus*)	Gallus_gallus-5.0	Roundworm (*Caenorhabditis elegans*)	WBcel235	-minScore=5000 -linearGap=loose
Chicken (*Gallus gallus*)	Gallus_gallus-5.0	Starlet sea anemone (*Nematostella vectensis*)	ASM20922v1	-minScore=5000 -linearGap=loose
Chicken (*Gallus gallus*)	Gallus_gallus-5.0	Choanoflagellate (*Salpingoeca rosetta*)	Proterospongia_ sp_ATCC50818	-minScore=5000 -linearGap=loose
Medaka (*Oryzias latipes*)	HdrR	Amazon molly (*Poecilia formosa*)	PoeFor_5.1.2	-minScore=3000 -linearGap=medium
Medaka (*Oryzias latipes*)	HdrR	Tilapia (*Oreochromis niloticus*)	Orenil1.0	-minScore=3000 -linearGap=medium
Medaka (*Oryzias latipes*)	HdrR	Fugu (*Takifugu rubripes*)	FUGU 4.0	-minScore=3000 -linearGap=medium
Medaka (*Oryzias latipes*)	HdrR	Mudskipper (*Boleophthalmus pectinirostris*)	BP	-minScore=3000 -linearGap=medium
Medaka (*Oryzias latipes*)	HdrR	Zebrafish (*Danio rerio*)	GRCz10	-minScore=3000 -linearGap=medium
Medaka (*Oryzias latipes*)	HdrR	Spotted gar (*Lepisosteus oculatus*)	LepOcu1	-minScore=3000 -linearGap=medium
Medaka (*Oryzias latipes*)	HdrR	Coelacanth (*Latimeria chalumnae*)	LatCha1	-minScore=5000 -linearGap=loose
Medaka (*Oryzias latipes*)	HdrR	Elephant shark (*Callorhinchus milii*)	calMil1	-minScore=5000 -linearGap=loose
Medaka (*Oryzias latipes*)	HdrR	Lamprey (*Petromyzon marinus*)	Pmarinus_7.0	-minScore=5000 -linearGap=loose
Medaka (*Oryzias latipes*)	HdrR	Vase tunicate (*Ciona intestinalis*)	KH	-minScore=5000 -linearGap=loose
Medaka (*Oryzias latipes*)	HdrR	Amphioxus (*Branchiostoma floridae*)	v1.0	-minScore=5000 -linearGap=loose
Medaka (*Oryzias latipes*)	HdrR	Roundworm (*Caenorhabditis elegans*)	WBcel235	-minScore=5000 -linearGap=loose
Medaka (*Oryzias latipes*)	HdrR	Starlet sea anemone (*Nematostella vectensis*)	ASM20922v1	-minScore=5000 -linearGap=loose
Medaka (*Oryzias latipes*)	HdrR	Choanoflagellate (*Salpingoeca rosetta*)	Proterospongia_ sp_ATCC50818	-minScore=5000 -linearGap=loose

Table S7. Statistical information in Figure 3.

		Kruskal–Wallis rank sum test			
**Species**	**Category**	**Test statistic (H)**	**Degrees of freedom**	***P* value**	**FDR**
Mouse	Mouse	17.0	7	0.0175	0.0191
	Human	17.8	7	0.0128	0.0191
	Cow	16.6	7	0.0204	0.0204
	Opossum	18.8	7	0.0088	0.0175
	Chicken	20.4	7	0.0047	0.0150
	Western clawed frog	20.2	7	0.0052	0.0150
	Coelacanth	20.4	7	0.0048	0.0150
	Spotted gar	18.1	7	0.0113	0.0191
	Elephant shark	19.7	7	0.0063	0.0150
	Lamprey	17.4	7	0.0150	0.0191
	Vase tunicate	17.1	7	0.0170	0.0191
	Amphioxus	20.7	7	0.0043	0.0150
Chicken	Chicken	18.2	7	0.0110	0.0130
	Turkey	14.5	7	0.0429	0.0429
	American alligator	21.7	7	0.0029	0.0054
	Softshell turtle	20.8	7	0.0041	0.0067
	Anole lizard	21.7	7	0.0028	0.0054
	Mouse	22.3	7	0.0023	0.0054
	Western clawed frog	22.0	7	0.0026	0.0054
	Coelacanth	21.7	7	0.0029	0.0054
	Spotted gar	22.0	7	0.0025	0.0054
	Elephant shark	21.9	7	0.0027	0.0054
	Lamprey	17.4	7	0.0149	0.0161
	Vase tunicate	19.1	7	0.0079	0.0103
	Amphioxus	19.6	7	0.0065	0.0094
Medaka	Medaka	16.7	6	0.0105	0.0144
	Amazon molly	18	6	0.0063	0.0124
	Tilapia	17.8	6	0.0068	0.0124
	Fugu	17.4	6	0.0079	0.0124
	Zebrafish	16	6	0.0139	0.0170
	Spotted gar	14.5	6	0.0244	0.0268
	Coelacanth	17.7	6	0.0070	0.0124
	Elephant shark	19.6	6	0.0032	0.0124
	Lamprey	18	6	0.0062	0.0124
	Vase tunicate	8.85	6	0.1820	0.1820
	Amphioxus	19.6	6	0.0032	0.0124

Table S8. Statistical information in Figure 4.

Species	Category	Kruskal–Wallis rank sum test			
		**Test statistic (H)**	**Degrees of freedom**	***P* value**	**FDR**
Mouse	Mouse	36.7	16	0.0023	0.0252
	Human	24.3	16	0.0836	0.0912
	Cow	28.2	16	0.0302	0.0497
	Opossum	27.2	16	0.0390	0.0520
	Chicken	27.8	16	0.0331	0.0497
	Western clawed frog	25.5	16	0.0622	0.0746
	Coelacanth	33.5	16	0.0063	0.0252
	Spotted gar	32.2	16	0.0094	0.0282
	Elephant shark	30.3	16	0.0165	0.0396
	Lamprey	19.8	16	0.2300	0.2300
	Vase tunicate	29.1	16	0.0234	0.0468
	Amphioxus	33.7	16	0.0059	0.0252
Chicken	Chicken	15.6	12	0.2110	0.2286
	Turkey	22.4	12	0.0333	0.1092
	American alligator	21.6	12	0.0420	0.1092
	Softshell turtle	23.0	12	0.0278	0.1092
	Anole lizard	22.1	12	0.0366	0.1092
	Mouse	16.5	12	0.1680	0.2184
	Western clawed frog	15.8	12	0.2000	0.2286
	Coelacanth	19.4	12	0.0793	0.1289
	Spotted gar	20.2	12	0.0630	0.1289
	Elephant shark	22.5	12	0.0320	0.1092
	Lamprey	12.2	12	0.4320	0.4320
	Vase tunicate	19.7	12	0.0738	0.1289
	Amphioxus	18.0	12	0.1160	0.1676
Medaka	Medaka	20.8	15	0.1440	0.1760
	Amazon molly	30.1	15	0.0117	0.0429
	Tilapia	21.5	15	0.1220	0.1678
	Fugu	22.7	15	0.0897	0.1645
	Zebrafish	21.5	15	0.1200	0.1678
	Spotted gar	18.3	15	0.2450	0.2450
	Coelacanth	27.7	15	0.0237	0.0652
	Elephant shark	43.1	15	0.0001	0.0011
	Lamprey	25.2	15	0.0476	0.1047
	Vase tunicate	19.9	15	0.1760	0.1936
	Amphioxus	31.7	15	0.0072	0.0396

Table S9. Detailed information on representative enhancers from the VISTA Enhancer Database.

Enhancer ID	Genomic position (GRCm38)	Flanking genes	Expression pattern
mm3	12:111142738–111143613	*Rcor1;*<br>*Traf3*	neuraltube[4/4];<br>hindbrain(rhombencephalon)[4/4];<br>forebrain[4/4]
mm7	14:61004739–61005741	*Tnfrsf19*	forebrain[6/6]
mm9	8:46770571–46772246	*Irf2*	eye[3/4]
mm109	18:56896918–56898991	*March3*	heart[3/11]
mm257	18:75502363–75503667	*Ctif*	midbrain(mesencephalon)[6/8];<br>heart[8/8]
mm303	9:103679109–103679657	*4932413F04Rik;*<br>*Nphp3*	heart[3/6]
mm324	19:47908459–47910596	*Itprip*	nose[4/11]
mm394	5:24730554–24731843	*Wdr86;*<br>*Crygn*	midbrain(mesencephalon)[6/11];<br>eye[5/11]
mm422	2:4556144–4557875	*Frmd4a*	forebrain[4/7];<br>limb[4/7]
mm515	2:75422202–75424576	*Mtx2;*<br>*9430019J16Rik*	neuraltube[4/7];<br>hindbrain(rhombencephalon)[5/7];<br>forebrain[4/7];<br>limb[5/7]
mm622	5:111601262–111602739	*C130026L21Rik;*<br>*E130006D01Rik*	branchialarch[9/11]
mm790	1:127554365–127556879	*Tmem163*	neuraltube[3/3];<br>hindbrain(rhombencephalon)[3/3];<br>midbrain(mesencephalon)[3/3];<br>forebrain[3/3]

Table S10. Statistical information in Figure S7.

		Kruskal–Wallis rank sum test			
**Species**	**Category**	**Test statistic (H)**	**Degrees of freedom**	***P* value**	**FDR**
Mouse	Mouse	36.7	16	0.0023	0.0275
	Human	24.8	16	0.0743	0.0991
	Cow	30.4	16	0.0160	0.0410
	Opossum	29.5	16	0.0206	0.0412
	Chicken	27.8	16	0.0331	0.0567
	Western clawed frog	23.6	16	0.0989	0.1187
	Coelacanth	34.3	16	0.0050	0.0298
	Spotted gar	20.8	16	0.1870	0.2040
	Elephant shark	27.3	16	0.0384	0.0576
	Lamprey	14.9	16	0.5320	0.5320
	Vase tunicate	30.2	16	0.0171	0.0410
	Amphioxus	32.5	16	0.0087	0.0348
Chicken	Chicken	15.5	12	0.2160	0.2470
	Turkey	22.2	12	0.0349	0.1144
	American alligator	21.6	12	0.0420	0.1144
	Softshell turtle	22.1	12	0.0366	0.1144
	Anole lizard	20.3	12	0.0618	0.1144
	Mouse	14.9	12	0.2470	0.2470
	Western clawed frog	21.2	12	0.0471	0.1144
	Coelacanth	18.3	12	0.1080	0.1560
	Spotted gar	19.8	12	0.0704	0.1144
	Elephant shark	19.9	12	0.0687	0.1144
	Lamprey	14.9	12	0.2460	0.2470
	Vase tunicate	20.4	12	0.0603	0.1144
	Amphioxus	16.5	12	0.1680	0.2184
Medaka	Medaka	18.9	15	0.2170	0.2170
	Amazon molly	27.4	15	0.0257	0.0707
	Tilapia	23.8	15	0.0691	0.1267
	Fugu	21.5	15	0.1220	0.1678
	Zebrafish	19.1	15	0.2110	0.2170
	Spotted gar	22	15	0.1080	0.1678
	Coelacanth	20.9	15	0.1390	0.1699
	Elephant shark	43.7	15	0.0001	0.0013
	Lamprey	24.7	15	0.0535	0.1177
	Vase tunicate	32.1	15	0.0063	0.0231
	Amphioxus	32.3	15	0.0059	0.0231

Table S11. Statistical information in Figure S8a.

		Kruskal–Wallis rank sum test			
**Species**	**Category**	**Test statistic (H)**	**Degrees of freedom**	***P* value**	**FDR**
Mouse	Mouse	17.0	7	0.0175	0.0210
	Human	17.9	7	0.0124	0.0201
	Cow	16.6	7	0.0022	0.0178
	Opossum	19.3	7	0.0074	0.0178
	Chicken	19.4	7	0.0071	0.0178
	Western clawed frog	15.1	7	0.0350	0.0382
	Coelacanth	19.8	7	0.0060	0.0178
	Spotted gar	12.5	7	0.0847	0.0847
	Elephant shark	18.6	7	0.0097	0.0194
	Lamprey	17.4	7	0.0151	0.0201
	Vase tunicate	17.5	7	0.0147	0.0201
	Amphioxus	20.6	7	0.0043	0.0178
Chicken	Chicken	19.8	7	0.0061	0.0079
	Turkey	19.0	7	0.0082	0.0089
	American alligator	22.0	7	0.0026	0.0055
	Softshell turtle	21.3	7	0.0034	0.0055
	Anole lizard	21.4	7	0.0033	0.0055
	Mouse	21.3	7	0.0034	0.0055
	Western clawed frog	21.2	7	0.0035	0.0055
	Coelacanth	21.6	7	0.0029	0.0055
	Spotted gar	21.2	7	0.0035	0.0055
	Elephant shark	22.2	7	0.0024	0.0055
	Lamprey	17.5	7	0.0143	0.0143
	Vase tunicate	19.1	7	0.0077	0.0089
	Amphioxus	21.0	7	0.0038	0.0055
Medaka	Medaka	17.3	6	0.0083	0.0156
	Amazon molly	16.8	6	0.0099	0.0156
	Tilapia	17.5	6	0.0075	0.0156
	Fugu	17.1	6	0.0088	0.0156
	Zebrafish	15.7	6	0.0154	0.0212
	Spotted gar	14	6	0.0297	0.0363
	Coelacanth	19.5	6	0.0034	0.0136
	Elephant shark	19.6	6	0.0032	0.0136
	Lamprey	9.85	6	0.1310	0.1441
	Vase tunicate	8.76	6	0.1870	0.1870
	Amphioxus	19.3	6	0.0037	0.0136

Table S12. Statistical information in Figure S8b.

		Kruskal–Wallis rank sum test			
**Species**	**Category**	**Test statistic (H)**	**Degrees of freedom**	***P* value**	**FDR**
Mouse	Mouse	17.1	7	0.0167	0.0182
	Human	17.1	7	0.0167	0.0182
	Cow	17.1	7	0.0167	0.0182
	Opossum	18.5	7	0.0099	0.0164
	Chicken	20.2	7	0.0051	0.0146
	Western clawed frog	18.5	7	0.0100	0.0164
	Coelacanth	20.2	7	0.0051	0.0146
	Spotted gar	20.2	7	0.0051	0.0146
	Elephant shark	19.7	7	0.0061	0.0146
	Lamprey	18.3	7	0.0109	0.0164
	Vase tunicate	15.6	7	0.0286	0.0286
	Amphioxus	20.9	7	0.0039	0.0146
Chicken	Chicken	18.8	7	0.0087	0.0126
	Turkey	17.7	7	0.0136	0.0153
	American alligator	22.7	7	0.0019	0.0056
	Softshell turtle	20.5	7	0.0046	0.0075
	Anole lizard	21.7	7	0.0029	0.0056
	Mouse	22.4	7	0.0022	0.0056
	Western clawed frog	21.6	7	0.0030	0.0056
	Coelacanth	21.9	7	0.0026	0.0056
	Spotted gar	21.6	7	0.0030	0.0056
	Elephant shark	21.7	7	0.0028	0.0056
	Lamprey	17.6	7	0.0141	0.0153
	Vase tunicate	18.3	7	0.0108	0.0140
	Amphioxus	17.3	7	0.0154	0.0154
Medaka	Medaka	17.2	6	0.0087	0.0131
	Amazon molly	16.4	6	0.0119	0.0131
	Tilapia	18.1	6	0.0059	0.0131
	Fugu	17.2	6	0.0087	0.0131
	Zebrafish	16.5	6	0.0112	0.0131
	Spotted gar	13.7	6	0.0334	0.0334
	Coelacanth	16.7	6	0.0106	0.0131
	Elephant shark	19.6	6	0.0032	0.0131
	Lamprey	17.6	6	0.0072	0.0131
	Vase tunicate	19	6	0.0041	0.0131
	Amphioxus	19.6	6	0.0032	0.0131

Table S13. Statistical information in Figure S8c.

		Kruskal–Wallis rank sum test			
**Species**	**Category**	**Test statistic (H)**	**Degrees of freedom**	***P* value**	**FDR**
Mouse	Mouse	17.4	7	0.0150	0.0200
	Human	17.4	7	0.0148	0.0200
	Cow	16.6	7	0.0204	0.0245
	Opossum	18.5	7	0.0098	0.0200
	Chicken	18.9	7	0.0086	0.0200
	Western clawed frog	20.5	7	0.0047	0.0200
	Coelacanth	19.6	7	0.0066	0.0200
	Spotted gar	13.1	7	0.0705	0.0705
	Elephant shark	17.8	7	0.0130	0.0200
	Lamprey	18.3	7	0.0109	0.0200
	Vase tunicate	16.1	7	0.0243	0.0265
	Amphioxus	19.9	7	0.0058	0.0200
Chicken	Chicken	20.2	7	0.0052	0.0068
	Turkey	18.8	7	0.0087	0.0094
	American alligator	22.7	7	0.0019	0.0062
	Softshell turtle	20.7	7	0.0043	0.0062
	Anole lizard	21.1	7	0.0036	0.0062
	Mouse	21.5	7	0.0031	0.0062
	Western clawed frog	21.1	7	0.0036	0.0062
	Coelacanth	21.4	7	0.0033	0.0062
	Spotted gar	21.8	7	0.0028	0.0062
	Elephant shark	22.0	7	0.0025	0.0062
	Lamprey	18.0	7	0.0121	0.0121
	Vase tunicate	19.0	7	0.0081	0.0094
	Amphioxus	20.7	7	0.0042	0.0062
Medaka	Medaka	17.7	6	0.0069	0.0150
	Amazon molly	16.4	6	0.0117	0.0169
	Tilapia	17.7	6	0.0071	0.0150
	Fugu	17.3	6	0.0082	0.0150
	Zebrafish	16.3	6	0.0123	0.0169
	Spotted gar	15	6	0.0206	0.0252
	Coelacanth	19.6	6	0.0032	0.0143
	Elephant shark	19.4	6	0.0036	0.0143
	Lamprey	10.6	6	0.1030	0.1133
	Vase tunicate	8.81	6	0.1840	0.1840
	Amphioxus	19.1	6	0.0039	0.0143

Table S14. Statistical information in Figure S9.

		Kruskal–Wallis rank sum test			
**Species**	**Category**	**Test statistic (H)**	**Degrees of freedom**	***P* value**	**FDR**
Mouse	Mouse	22.3	7	0.0023	0.0119
	Rat	17.7	7	0.0136	0.0193
	Rabbit	16.2	7	0.0236	0.0267
	Human	14.3	7	0.0464	0.0464
	Cow	16.3	7	0.0225	0.0267
	Opossum	19.5	7	0.0067	0.0121
	Chicken	21.4	7	0.0032	0.0119
	Western clawed frog	20.8	7	0.0041	0.0119
	Coelacanth	21.3	7	0.0034	0.0119
	Spotted gar	19.0	7	0.0082	0.0127
	Elephant shark	20.7	7	0.0042	0.0119
	Lamprey	16.4	7	0.0220	0.0267
	Vase tunicate	15.7	7	0.0285	0.0303
	Amphioxus	19.4	7	0.0071	0.0121
	Roundworm	19.6	7	0.0064	0.0121
	Starlet sea anemone	19.9	7	0.0058	0.0121
	Choanoflagellates	21.7	7	0.0029	0.0119
Chicken	Chicken	18.4	7	0.0101	0.0114
	Turkey	20.4	7	0.0048	0.0077
	Zebra finch	12.3	7	0.0909	0.0909
	American alligator	20.9	7	0.0039	0.0077
	Softshell turtle	21.3	7	0.0033	0.0077
	Anole lizard	21.5	7	0.0031	0.0077
	Mouse	22.1	7	0.0024	0.0077
	Western clawed frog	22.5	7	0.0021	0.0077
	Coelacanth	21.9	7	0.0027	0.0077
	Spotted gar	21.5	7	0.0031	0.0077
	Elephant shark	22.1	7	0.0024	0.0077
	Lamprey	17.5	7	0.0147	0.0156
	Vase tunicate	18.6	7	0.0096	0.0114
	Amphioxus	20.1	7	0.0054	0.0077
	Roundworm	20.5	7	0.0046	0.0077
	Starlet sea anemone	18.9	7	0.0084	0.0110
	Choanoflagellates	20.3	7	0.0050	0.0077
Medaka	Medaka	16.7	6	0.0103	0.0129
	Amazon molly	18.0	6	0.0063	0.0102
	Tilapia	17.5	6	0.0075	0.0102
	Fugu	17.7	6	0.0070	0.0102
	Mudskipper	17.6	6	0.0074	0.0102
	Zebrafish	15.1	6	0.0198	0.0228
	Spotted gar	14.0	6	0.0295	0.0295
	Coelacanth	17.9	6	0.0065	0.0102
	Elephant shark	19.6	6	0.0032	0.0102
	Lamprey	17.6	6	0.0072	0.0102
	Vase tunicate	14.5	6	0.0244	0.0261
	Amphioxus	19.6	6	0.0032	0.0102
	Roundworm	17.7	6	0.0069	0.0102
	Starlet sea anemone	19.2	6	0.0039	0.0102
	Choanoflagellates	19.4	6	0.0036	0.0102

Table S15. Statistical information in Figure S10a.

		Kruskal–Wallis rank sum test			
**Species**	**Category**	**Test statistic (H)**	**Degrees of freedom**	***P* value**	**FDR**
Mouse	Mouse	21.0	7	0.0038	0.0062
	Human	15.9	7	0.0262	0.0314
	Cow	16.3	7	0.0227	0.0303
	Opossum	20.8	7	0.0041	0.0062
	Chicken	22.2	7	0.0024	0.0062
	Western clawed frog	21.6	7	0.0030	0.0062
	Coelacanth	22.0	7	0.0026	0.0062
	Spotted gar	21.2	7	0.0035	0.0062
	Elephant shark	22.3	7	0.0022	0.0062
	Lamprey	15.2	7	0.0335	0.0335
	Vase tunicate	15.4	7	0.0314	0.0335
	Amphioxus	21.9	7	0.0026	0.0062
Chicken	Chicken	16.5	7	0.0213	0.0231
	Turkey	13.7	7	0.0561	0.0561
	American alligator	21.7	7	0.0029	0.0054
	Softshell turtle	20.8	7	0.0041	0.0067
	Anole lizard	21.7	7	0.0028	0.0054
	Mouse	22.2	7	0.0024	0.0054
	Western clawed frog	22.1	7	0.0025	0.0054
	Coelacanth	21.7	7	0.0029	0.0054
	Spotted gar	22.0	7	0.0026	0.0054
	Elephant shark	21.8	7	0.0028	0.0054
	Lamprey	18.2	7	0.0112	0.0146
	Vase tunicate	19.1	7	0.0079	0.0114
	Amphioxus	16.6	7	0.0200	0.0231
Medaka	Medaka	18.4	6	0.0054	0.0074
	Amazon molly	19.3	6	0.0037	0.0074
	Tilapia	19.1	6	0.0040	0.0074
	Fugu	18.6	6	0.0049	0.0074
	Zebrafish	17.7	6	0.0069	0.0084
	Spotted gar	17.0	6	0.0095	0.0105
	Coelacanth	19.3	6	0.0037	0.0074
	Elephant shark	19.4	6	0.0036	0.0074
	Lamprey	18.7	6	0.0048	0.0074
	Vase tunicate	10.2	6	0.1170	0.1170
	Amphioxus	19.1	6	0.0039	0.0074

Table S16. Statistical information in Figure S10b.

		Kruskal–Wallis rank sum test			
**Species**	**Category**	**Test statistic (H)**	**Degrees of freedom**	***P* value**	**FDR**
Mouse	Mouse	22.0	7	0.0026	0.0048
	Human	18.1	7	0.0114	0.0152
	Cow	17.5	7	0.0145	0.0174
	Opossum	21.3	7	0.0033	0.0050
	Chicken	22.4	7	0.0021	0.0048
	Western clawed frog	21.9	7	0.0027	0.0048
	Coelacanth	21.9	7	0.0026	0.0048
	Spotted gar	21.8	7	0.0028	0.0048
	Elephant shark	22.1	7	0.0024	0.0048
	Lamprey	15.5	7	0.0305	0.0305
	Vase tunicate	15.8	7	0.0270	0.0295
	Amphioxus	22.4	7	0.0022	0.0048
Chicken	Chicken	20.1	7	0.0055	0.0065
	Turkey	21.7	7	0.0028	0.0046
	American alligator	22.5	7	0.0021	0.0046
	Softshell turtle	22.0	7	0.0025	0.0046
	Anole lizard	22.4	7	0.0021	0.0046
	Mouse	22.6	7	0.0020	0.0046
	Western clawed frog	22.7	7	0.0019	0.0046
	Coelacanth	22.0	7	0.0025	0.0046
	Spotted gar	21.5	7	0.0032	0.0046
	Elephant shark	21.9	7	0.0027	0.0046
	Lamprey	19.6	7	0.0065	0.0070
	Vase tunicate	13.6	7	0.0585	0.0585
	Amphioxus	20.7	7	0.0042	0.0055
Medaka	Medaka	16.4	6	0.0118	0.0130
	Amazon molly	17.7	6	0.0071	0.0100
	Tilapia	18.2	6	0.0058	0.0100
	Fugu	17.6	6	0.0073	0.0100
	Zebrafish	16.5	6	0.0113	0.0130
	Spotted gar	14.0	6	0.0297	0.0297
	Coelacanth	18.1	6	0.0060	0.0100
	Elephant shark	19.6	6	0.0032	0.0094
	Lamprey	19.5	6	0.0034	0.0094
	Vase tunicate	19.5	6	0.0034	0.0094
	Amphioxus	19.6	6	0.0032	0.0094

Table S17. Statistical information in Figure S10c.

		Kruskal–Wallis rank sum test			
**Species**	**Category**	**Test statistic (H)**	**Degrees of freedom**	***P* value**	**FDR**
Mouse	Mouse	17.1	7	0.0170	0.0227
	Human	16.4	7	0.0216	0.0236
	Cow	17.8	7	0.0130	0.0195
	Opossum	19.9	7	0.0058	0.0116
	Chicken	21.3	7	0.0033	0.0116
	Western clawed frog	20.4	7	0.0048	0.0116
	Coelacanth	19.9	7	0.0058	0.0116
	Spotted gar	18.8	7	0.0088	0.0151
	Elephant shark	20.2	7	0.0051	0.0116
	Lamprey	16.6	7	0.0202	0.0236
	Vase tunicate	15.8	7	0.0267	0.0267
	Amphioxus	21.3	7	0.0034	0.0116
Chicken	Chicken	18.9	7	0.0084	0.0102
	Turkey	16.7	7	0.0194	0.0194
	American alligator	21.7	7	0.0029	0.0058
	Softshell turtle	21.1	7	0.0037	0.0060
	Anole lizard	21.6	7	0.0030	0.0058
	Mouse	22.1	7	0.0025	0.0058
	Western clawed frog	22.0	7	0.0026	0.0058
	Coelacanth	21.7	7	0.0029	0.0058
	Spotted gar	21.5	7	0.0031	0.0058
	Elephant shark	21.9	7	0.0026	0.0058
	Lamprey	17.7	7	0.0132	0.0143
	Vase tunicate	19.3	7	0.0072	0.0102
	Amphioxus	18.9	7	0.0086	0.0102
Medaka	Medaka	17.0	6	0.0093	0.0128
	Amazon molly	18.4	6	0.0053	0.0121
	Tilapia	17.9	6	0.0066	0.0121
	Fugu	17.0	6	0.0092	0.0128
	Zebrafish	16.0	6	0.0138	0.0169
	Spotted gar	12.4	6	0.0533	0.0586
	Coelacanth	18.1	6	0.0060	0.0121
	Elephant shark	19.6	6	0.0032	0.0121
	Lamprey	18.5	6	0.0052	0.0121
	Vase tunicate	11.2	6	0.0838	0.0838
	Amphioxus	19.6	6	0.0032	0.0121

Table S18. Statistical information in Figure S11.

		Kruskal–Wallis rank sum test			
**Species**	**Category**	**Test statistic (H)**	**Degrees of freedom**	***P* value**	**FDR**
Mouse	Mouse	28.1	11	0.0031	0.0036
	Human	28.0	11	0.0032	0.0036
	Cow	29.0	11	0.0023	0.0035
	Opossum	29.7	11	0.0018	0.0034
	Chicken	30.4	11	0.0014	0.0034
	Western clawed frog	30.2	11	0.0015	0.0034
	Coelacanth	30.1	11	0.0015	0.0034
	Spotted gar	30.2	11	0.0015	0.0034
	Elephant shark	29.4	11	0.0020	0.0034
	Lamprey	27.9	11	0.0033	0.0036
	Vase tunicate	27.7	11	0.0036	0.0036
	Amphioxus	30.3	11	0.0014	0.0034

Table S19. Statistical information in Figure S12a.

		Kruskal–Wallis rank sum test			
**Species**	**Category**	**Test statistic (H)**	**Degrees of freedom**	***P* value**	**FDR**
Mouse	Mouse	19.8	7	0.0061	0.0145
	Human	18.8	7	0.0088	0.0149
	Cow	16.1	7	0.0243	0.0292
	Opossum	16.3	7	0.0225	0.0292
	Chicken	15.6	7	0.0290	0.0316
	Western clawed frog	19.3	7	0.0072	0.0145
	Coelacanth	15.0	7	0.0357	0.0357
	Spotted gar	18.5	7	0.0099	0.0149
	Elephant shark	20.0	7	0.0055	0.0145
	Lamprey	19.5	7	0.0068	0.0145
	Vase tunicate	20.3	7	0.0051	0.0145
	Amphioxus	19.4	7	0.0071	0.0145
Chicken	Chicken	20.1	7	0.0053	0.0106
	Turkey	19.9	7	0.0059	0.0106
	American alligator	20.2	7	0.0052	0.0106
	Softshell turtle	21.7	7	0.0028	0.0106
	Anole lizard	19.0	7	0.0081	0.0106
	Mouse	19.5	7	0.0068	0.0106
	Western clawed frog	17.4	7	0.0150	0.0163
	Coelacanth	21.5	7	0.0031	0.0106
	Spotted gar	19.0	7	0.0081	0.0106
	Elephant shark	20.9	7	0.0039	0.0106
	Lamprey	21.3	7	0.0034	0.0106
	Vase tunicate	17.4	7	0.0150	0.0163
	Amphioxus	15.4	7	0.0317	0.0317
Medaka	Medaka	17.8	6	0.0067	0.0067
	Amazon molly	18.7	6	0.0047	0.0052
	Tilapia	19.2	6	0.0039	0.0051
	Fugu	19.3	6	0.0036	0.0051
	Zebrafish	19.6	6	0.0032	0.0051
	Spotted gar	19.4	6	0.0036	0.0051
	Coelacanth	19.5	6	0.0034	0.0051
	Elephant shark	19.4	6	0.0036	0.0051
	Lamprey	19.4	6	0.0036	0.0051
	Vase tunicate	19.0	6	0.0042	0.0051
	Amphioxus	19.5	6	0.0034	0.0051

Table S20. Statistical information in Figure S12b.

		Kruskal–Wallis rank sum test			
**Species**	**Category**	**Test statistic (H)**	**Degrees of freedom**	***P* value**	**FDR**
Mouse	Mouse	18.7	7	0.0091	0.0144
	Human	19.4	7	0.0071	0.0144
	Cow	19.1	7	0.0078	0.0144
	Opossum	19.7	7	0.0064	0.0144
	Chicken	19.5	7	0.0066	0.0144
	Western clawed frog	20.2	7	0.0052	0.0144
	Coelacanth	13.5	7	0.0612	0.0612
	Spotted gar	16.6	7	0.0204	0.0223
	Elephant shark	18.6	7	0.0096	0.0144
	Lamprey	16.7	7	0.0194	0.0223
	Vase tunicate	17.1	7	0.0170	0.0223
	Amphioxus	21.0	7	0.0037	0.0144
Chicken	Chicken	16.0	7	0.0252	0.0252
	Turkey	18.4	7	0.0101	0.0146
	American alligator	21.6	7	0.0030	0.0108
	Softshell turtle	21.7	7	0.0029	0.0108
	Anole lizard	22.0	7	0.0025	0.0108
	Mouse	21.3	7	0.0033	0.0108
	Western clawed frog	17.9	7	0.0125	0.0163
	Coelacanth	18.5	7	0.0097	0.0146
	Spotted gar	20.3	7	0.0050	0.0108
	Elephant shark	16.5	7	0.0212	0.0230
	Lamprey	17.4	7	0.0150	0.0177
	Vase tunicate	19.1	7	0.0079	0.0146
	Amphioxus	20.3	7	0.0050	0.0108
Medaka	Medaka	17	6	0.0094	0.0207
	Amazon molly	18	6	0.0062	0.0207
	Tilapia	17.1	6	0.0088	0.0207
	Fugu	16.4	6	0.0116	0.0213
	Zebrafish	11.3	6	0.0798	0.0975
	Spotted gar	9.16	6	0.1650	0.1815
	Coelacanth	14.4	6	0.0252	0.0347
	Elephant shark	19.6	6	0.0032	0.0177
	Lamprey	14.8	6	0.0220	0.0346
	Vase tunicate	8.76	6	0.1870	0.1870
	Amphioxus	19.6	6	0.0032	0.0177

Table S21. Statistical information in Figure S12c.

		Kruskal–Wallis rank sum test			
**Species**	**Category**	**Test statistic (H)**	**Degrees of freedom**	***P* value**	**FDR**
Mouse	Mouse	19.9	7	0.0059	0.0088
	Human	16.1	7	0.0245	0.0267
	Cow	15.0	7	0.0357	0.0357
	Opossum	21.2	7	0.0034	0.0059
	Chicken	22.2	7	0.0024	0.0052
	Western clawed frog	22.4	7	0.0022	0.0052
	Coelacanth	21.9	7	0.0026	0.0052
	Spotted gar	21.9	7	0.0026	0.0052
	Elephant shark	22.2	7	0.0023	0.0052
	Lamprey	18.3	7	0.0109	0.0145
	Vase tunicate	17.5	7	0.0147	0.0176
	Amphioxus	22.3	7	0.0023	0.0052
Chicken	Chicken	20.5	7	0.0046	0.0075
	Turkey	18.0	7	0.0121	0.0143
	American alligator	20.7	7	0.0046	0.0075
	Softshell turtle	21.2	7	0.0035	0.0075
	Anole lizard	21.5	7	0.0081	0.0117
	Mouse	22.0	7	0.0025	0.0075
	Western clawed frog	22.1	7	0.0024	0.0075
	Coelacanth	21.8	7	0.0027	0.0075
	Spotted gar	21.6	7	0.0030	0.0075
	Elephant shark	21.6	7	0.0030	0.0075
	Lamprey	18.6	7	0.0096	0.0125
	Vase tunicate	13.6	7	0.0588	0.0588
	Amphioxus	17.6	7	0.0137	0.0148
Medaka	Medaka	16.6	6	0.0111	0.0111
	Amazon molly	17.8	6	0.0067	0.0104
	Tilapia	18.4	6	0.0053	0.0104
	Fugu	16.7	6	0.0085	0.0104
	Zebrafish	17.5	6	0.0063	0.0104
	Spotted gar	17.8	6	0.0079	0.0104
	Coelacanth	17.5	6	0.0057	0.0104
	Elephant shark	19.1	6	0.0042	0.0104
	Lamprey	19.3	6	0.0097	0.0106
	Vase tunicate	19	6	0.0044	0.0104
	Amphioxus	19.5	6	0.0049	0.0104

Table S22. Statistical information in Figure S13.

		Kruskal–Wallis rank sum test			
**Species**	**Category**	**Test statistic (H)**	**Degrees of freedom**	***P* value**	**FDR**
Mouse	Human	17.4	7	0.0149	0.0170
	Cow	17.6	7	0.0141	0.0170
	Opossum	21.5	7	0.0031	0.0073
	Chicken	21.7	7	0.0028	0.0073
	Western clawed frog	21.2	7	0.0035	0.0073
	Coelacanth	21.2	7	0.0034	0.0073
	Spotted gar	17.6	7	0.0141	0.0170
	Elephant shark	22.1	7	0.0025	0.0073
	Lamprey	17.1	7	0.0170	0.0170
	Vase tunicate	17.1	7	0.0168	0.0170
	Amphioxus	20.9	7	0.0040	0.0073
Chicken	Turkey	22.2	7	0.0024	0.0048
	American alligator	22.0	7	0.0025	0.0048
	Softshell turtle	21.0	7	0.0038	0.0051
	Anole lizard	21.5	7	0.0032	0.0048
	Mouse	21.9	7	0.0026	0.0048
	Western clawed frog	21.9	7	0.0026	0.0048
	Coelacanth	21.8	7	0.0027	0.0048
	Spotted gar	21.5	7	0.0031	0.0048
	Elephant shark	21.9	7	0.0027	0.0048
	Lamprey	17.4	7	0.0148	0.0148
	Vase tunicate	19.3	7	0.0074	0.0089
	Amphioxus	18.8	7	0.0087	0.0095
Medaka	Amazon molly	18.0	6	0.0062	0.0115
	Tilapia	18.5	6	0.0051	0.0115
	Fugu	17.7	6	0.0069	0.0115
	Zebrafish	16.5	6	0.0113	0.0141
	Spotted gar	14.9	6	0.0211	0.0234
	Coelacanth	18.3	6	0.0056	0.0115
	Elephant shark	19.6	6	0.0032	0.0115
	Lamprey	16.6	6	0.0110	0.0141
	Vase tunicate	9.1	6	0.1680	0.1680
	Amphioxus	19.6	6	0.0032	0.0115
