# Supplementary material for: Recapitulation-like developmental transitions of chromatin accessibility in vertebrates
Source: Zoological Lett. 2019 Nov 14;5:33. doi: 10.1186/s40851-019-0148-9 (PMC6857340; doi:10.1186/s40851-019-0148-9)
Supplement: Supplementary file 2 — Additional file 2 Figure S1. Identification of conserved mid-embryonic stages during medaka embryogenesis by transcriptome similarity. Figure S2. Genomic distribution of mouse, chicken, and medaka ACRs with respect to genome annotations. Figure S3. Enhancers that drive wider expression tend to have higher ATAC-seq signals. Figure S4. Four methods for estimating evolutionary ages of ACRs. Figure S5. Categorization of evolutionary ages of ACRs and expressed protein-coding genes did not differ among different methods. Figure S6. The recapitulative pattern was also observed for relative sequence length of evolutionarily categorized ACRs within the genome. Figure S7. Developmental gene expression levels did not show a recapitulative pattern in the analysis including genes lost secondarily, related to Fig. 4. Figure S8. The recapitulative pattern of whole-embryo chromatin accessibility was consistent between methods I, II, III, and IV, related to Fig. 3. Figure S9. Essentially the same recapitulative pattern was observed in the analysis using different sets of species. Figure S10. Essentially the same recapitulative pattern was observed for the analyses with different criteria in filtering ATAC-seq reads. Figure S11. Chromatin accessibility of mouse early stages did not show the recapitulative pattern. Figure S12. Exonic ACRs did not follow a recapitulative pattern. Figure S13. Similar recapitulative pattern observed for the chromatin accessibility under strong negative selection. Figure S14. No ACR could be detected at three of five representative regulatory regions associated with taxon-specific features. [file 40851_2019_148_MOESM2_ESM.pdf]

Additional file 2: Supplementary Figures

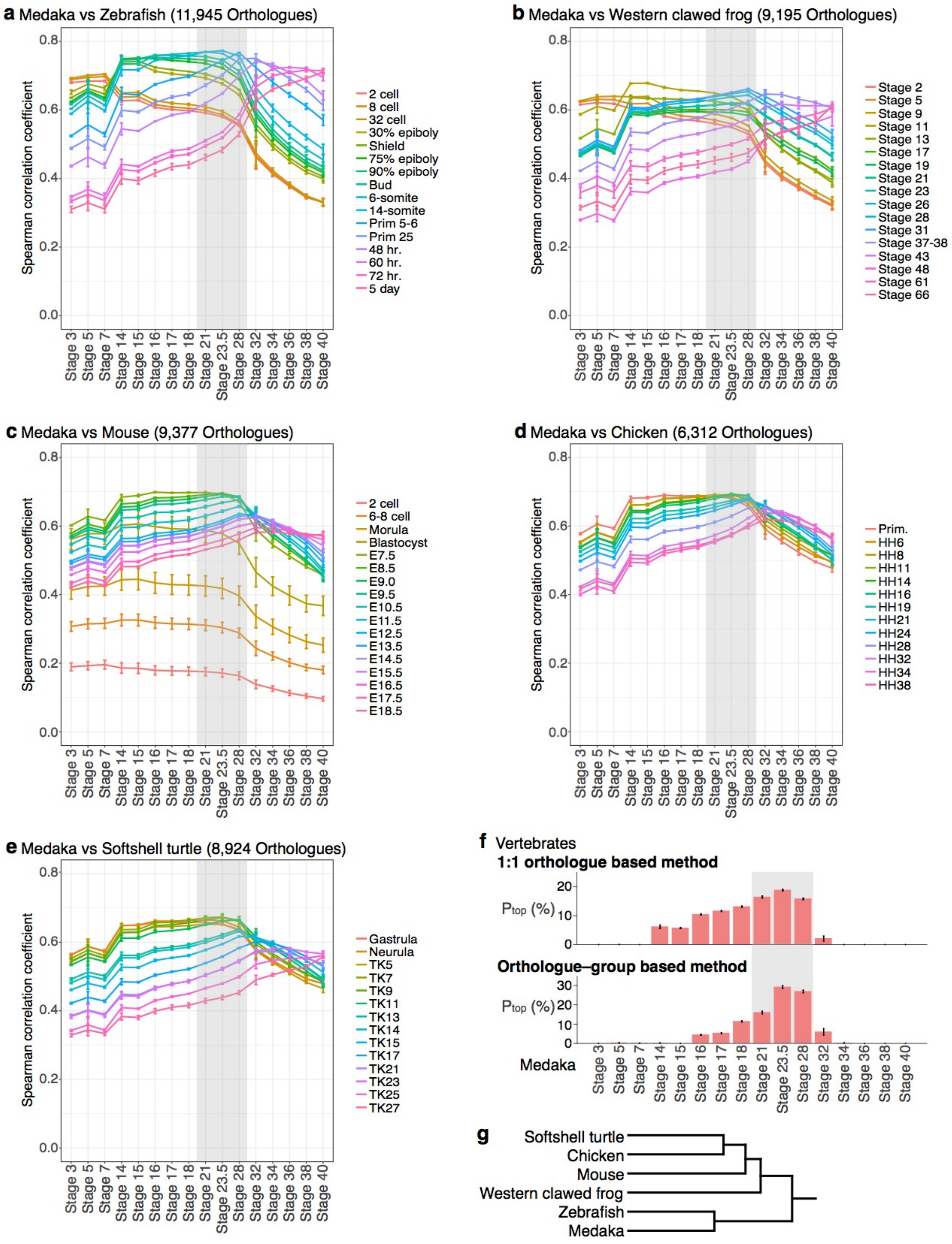

Figure S1. Identification of conserved mid-embryonic stages during medaka embryogenesis by transcriptome similarity. The developmental stages close to stage 24 of the medaka embryogenesis

(highlighted in gray) were highly conserved across the examined vertebrate species. **a–e** All- to all-stage comparisons of the transcriptome data of medaka embryos against zebrafish (**a**), western clawed frog (**b**), mouse (**c**), chicken (**d**), and softshell turtle (**e**) embryos are shown. The Spearman correlation coefficient was calculated by using FPKM values of orthologous genes to evaluate the transcriptomic similarity between samples. Lines were colored according to the developmental stages of each species. The number of orthologous genes used for comparing transcriptomes between species is shown at the top of each panel. For all line graphs, data are presented as mean ± 1 standard deviation of all pairs of biological replicates. **f** Conserved embryonic stages identified by 1:1 orthologue based method and by orthologue-group based method. The percentages of medaka developmental stages included in the most similar (lowest 1% of expDists) combinations of staged embryos from six vertebrates (mouse, chicken, softshell turtle, western clawed frog, zebrafish, and medaka) are shown as P_{top} (see Methods for details). Higher P_{top} values indicate developmental stages that are more highly conserved among the embryos of these vertebrates. expDist values were calculated to assess evolutionary conservation throughout the evolution of each of the six vertebrate species (see Additional file 3: Text S1 and Methods for details). To calculate expDists in the vertebrates, whole-embryo expression levels of 6,038 1:1 orthologues and 28,752 orthologue groups in the embryos of the six vertebrates were used for 1:1 orthologue based method and for orthologue-group based method, respectively [7,8,94] (see Additional file 3: Text S1 and Methods for details). Error bars represent the standard deviations for P_{top} values in 100 randomly selected biological replicates (BRI-exp). Changes in the P_{top} values of the developmental stages were statistically significant (Friedman test with 100 randomly selected BRI-exp for each species). Statistical information of the Friedman test is as follows: $\chi^2 = 1455.6$, df = 15, and $P < 2.2 \times 10^{-16}$ for 1:1 orthologue based method and $\chi^2 = 1409.5$, df = 15, and $P < 2.2 \times 10^{-16}$ for orthologue-group based method. **g** Phylogenetic relationships of the six vertebrate species referenced in calculating expDists.

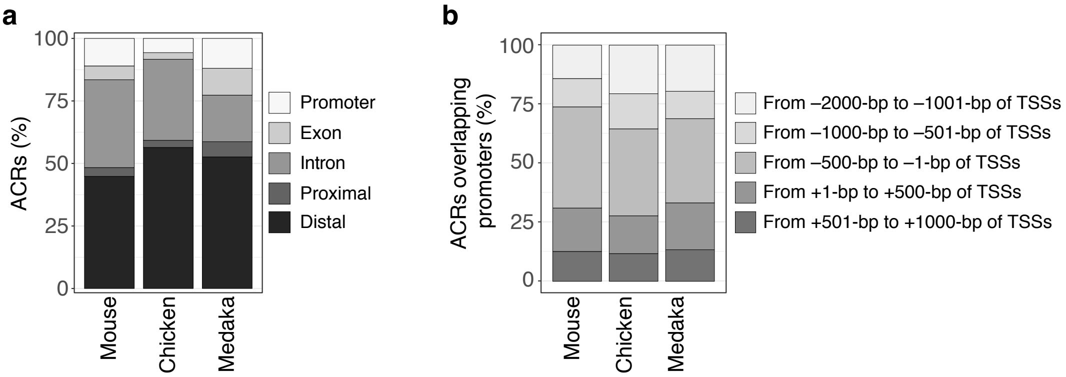

Figure S2. Genomic distribution of mouse, chicken, and medaka ACRs with respect to genome annotations. **a** Stacked bar graph shows the percentages of ACRs overlapping genomic regions with different annotations. ACRs in promoters were defined as those overlapping with regions between 2 kb upstream and 1 kb downstream of all transcription start sites (TSSs) of protein-coding genes. Proximal ACRs are defined as those within 5 kb upstream and 1 kb downstream of TSSs. ACRs in exons were defined as those overlapping with exons of protein-coding genes but not overlapping with promoters. ACRs in introns were defined as those in gene bodies of protein-coding genes but not overlapping with either exons or promoters. Proximal ACRs were defined as those overlapping with regions between 5 kb upstream and 1 kb downstream of all TSSs but not overlapping with either promoters or gene bodies. Distal ACRs are defined as the others not overlapping gene bodies. **b** The percentages of ACRs overlapping with promoters (at any of the developmental stages) according to the distances from the annotated TSSs are shown. Note that the genomic distributions of ACRs shown here were similar to those in previous studies [26,27].

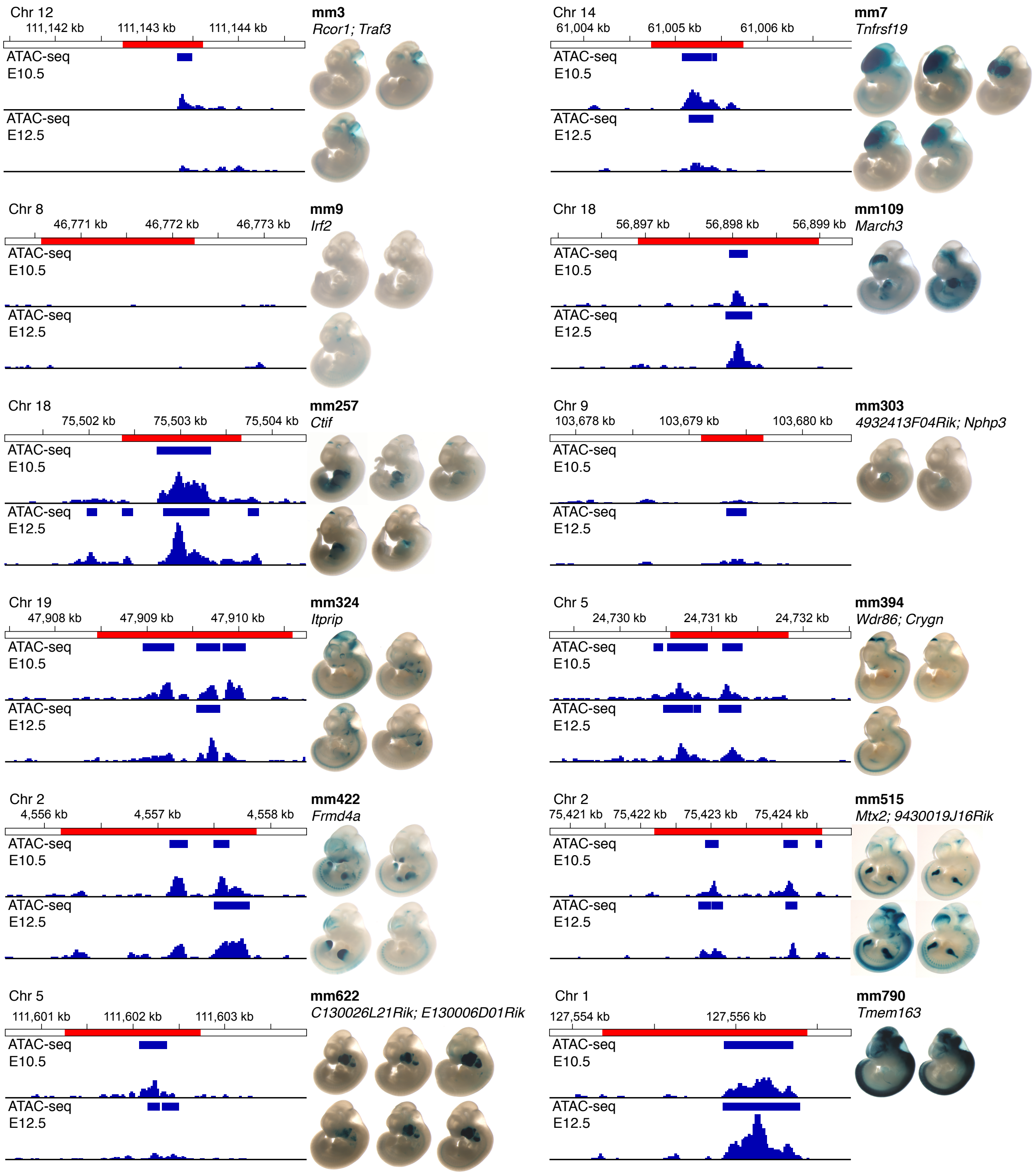

Figure S3. Enhancers that drive wider expression tend to have higher ATAC-seq signals. Enrichments of whole-embryo ATAC-seq reads in E10.5 and E12.5 mice and *in vivo* enhancer activity (*LacZ* reporter assay) in E11.5 mice at representative enhancers (red regions) from the VISTA Enhancer Database [23]. Images of embryos from the VISTA Enhancer Database [23] are shown with the VISTA Enhancer ID and the flanking genes. Enrichment of ATAC-seq reads shown in each panel represents the mean value of the three biological replicates. Deep blue boxes indicate the identified

ACRs. Detailed information on the annotated enhancers from the VISTA Enhancer Database [23] is given in Additional file 1: Table S9.

a Different methods to estimate evolutionary ages of ACRs

	ACRs consisting of multiple regions with different origins	Secondarily lost ACRs
**Method I**	Subdivided	Excluded
**Method II**	Subdivided	Included
**Method III**	Not subdivided	Excluded
**Method IV**	Not subdivided	Included

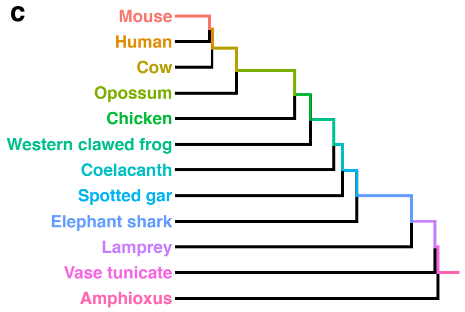

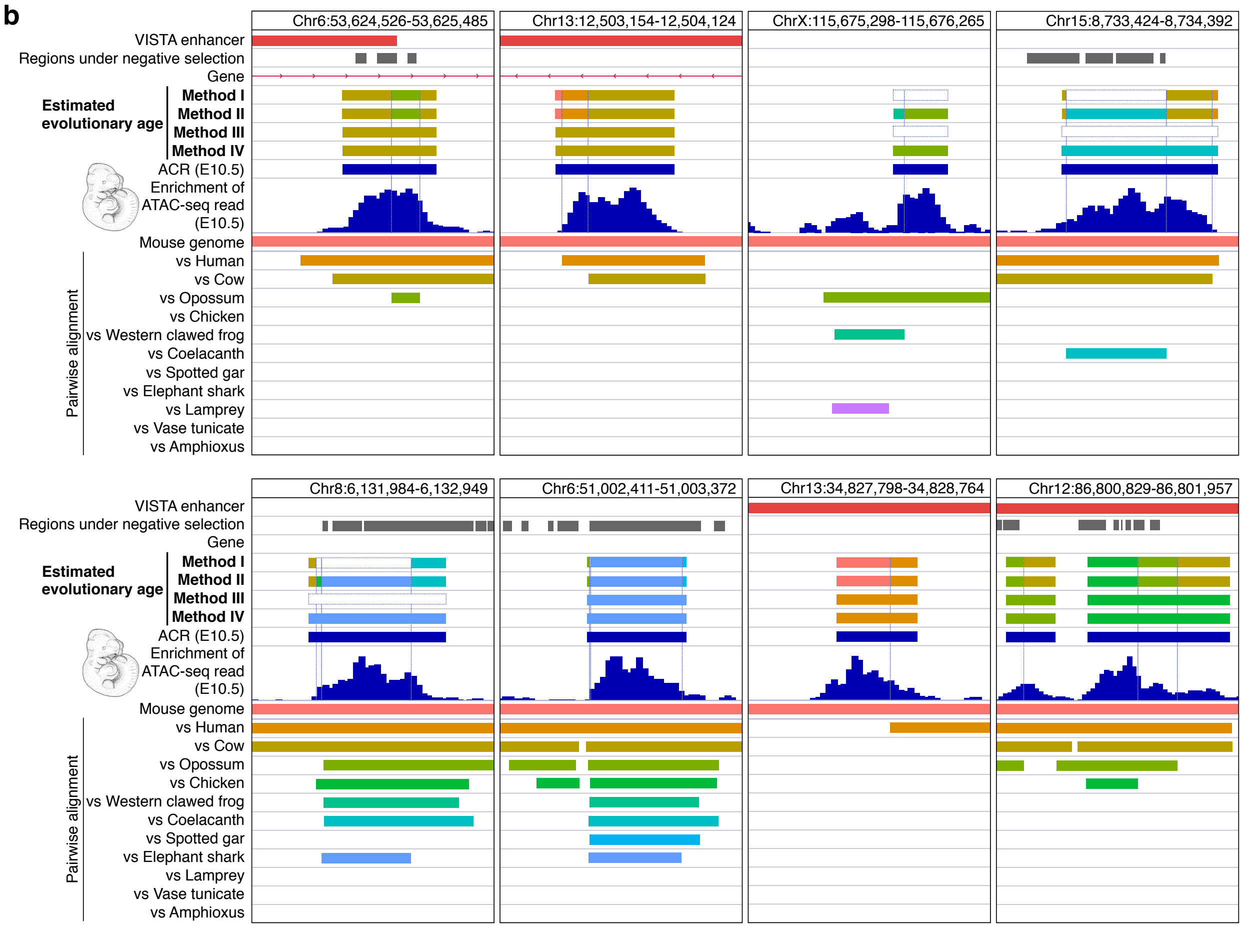

Figure S4. Four different methods for estimating evolutionary ages of ACRs. **a** Different methods used for estimating evolutionary ages of ACRs (methods I–IV). These four methods differ regarding whether the ACRs consisting of multiple regions with different evolutionary origins are subdivided into separate ACRs, and in whether the secondarily lost ACRs are excluded (see Methods for details). **b** Genome browser excerpts showing representative ACRs (deep blue boxes), their evolutionary ages estimated by the different methods, and the enrichments of ATAC-seq reads (deep blue signals). Evolutionary ages of ACRs were estimated by the four different methods (methods I–IV; see Methods for details) with whole-genome pairwise alignments between the different chordate species and the reference genomes. Colored regions in the tracks of "Pairwise alignment" represent aligned sequences of the reference genome to the corresponding species genome. Colors of ACRs in the track of

“Estimated evolutionary ages” indicate the estimated evolutionary ages. Each category includes ACRs that have originated during the corresponding-colored period in the evolutionary trajectory shown in **c**. **c** The phylogenetic tree of the species used for pairwise alignment.

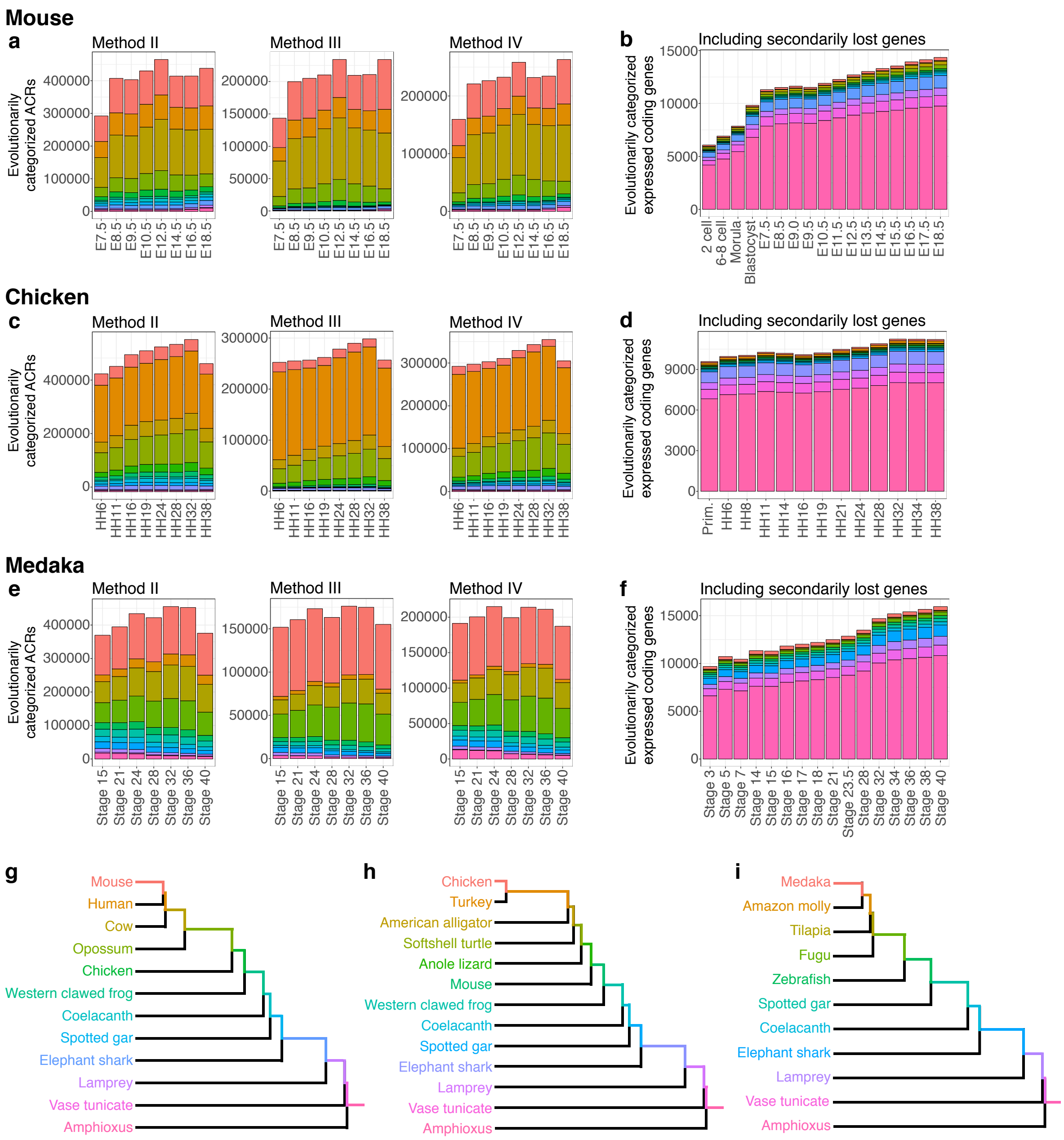

Figure S5. Categorization of evolutionary ages of ACRs and expressed protein-coding genes did not differ among different methods. In both cases of ACRs and expressed protein-coding genes, proportional distributions of their evolutionary ages were similar across the different methods (see also Figure 2). We also noted that almost all ACRs have been acquired since the origin of vertebrates, whereas most expressed protein-coding genes were acquired before that. Stacked bar graphs show the numbers of evolutionarily categorized ACRs and expressed protein-coding genes (FPKM > 1) at each developmental stage in mouse (**a**, **b**), chicken (**c**, **d**), and medaka (**e**, **f**). The evolutionary ages of ACRs were estimated based on Methods II–IV (see Methods and Additional file 2: Figure S4 for details). The evolutionary ages of protein-coding genes were estimated according to the most recent common ancestors of all the species sharing the similar sequences of the genes. In contrast to Figure 2, the expressed genes that were estimated to be lost secondarily in any of the aligned species are

included here (see Methods for details). Colors in each stacked bar graph indicate the categories of the evolutionary ages of each element. Each evolutionary category includes ACRs or expressed protein-coding genes that originated during the corresponding-colored period in the phylogenetic trees shown in **g** for mouse, **h** for chicken, and **i** for medaka.

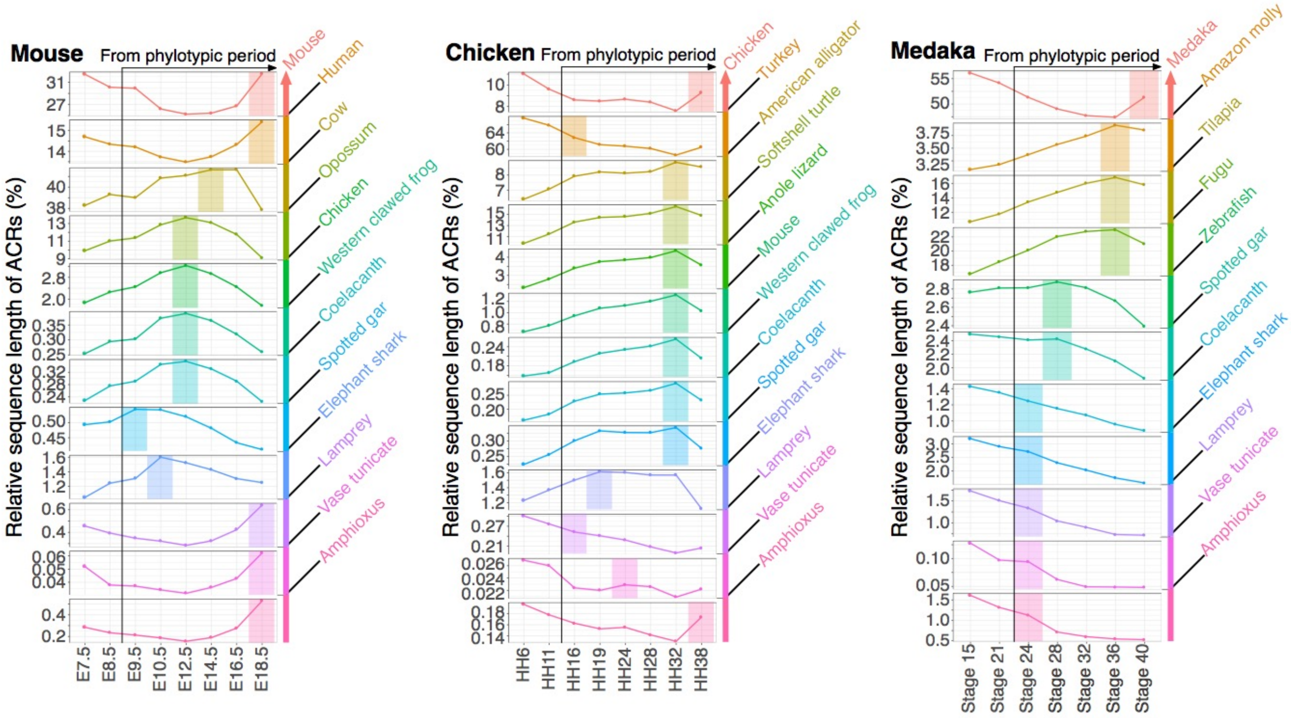

Figure S6. The recapitulative pattern was also observed for relative sequence length of evolutionarily categorized ACRs within the genome. The overall recapitulative pattern was also apparent for the genomic fractions of ACRs as well as for the developmental chromatin accessibility shown in Figure 3. The percentages corresponding to the summed length of evolutionarily categorized ACRs divided by the total length for all categories at developmental stages in three vertebrate species. The color of each category indicates the estimated evolutionary age of the region, as represented in the phylogenetic trees adjacent to the graphs. In each graph, the developmental stages with the highest value from the phylotypic period are highlighted in the corresponding color. Although the developmental stages with maximum chromatin accessibility of American alligator–turkey in the mid-to-late chicken embryogenesis did not follow the recapitulative pattern, the overall recapitulative pattern was observed.

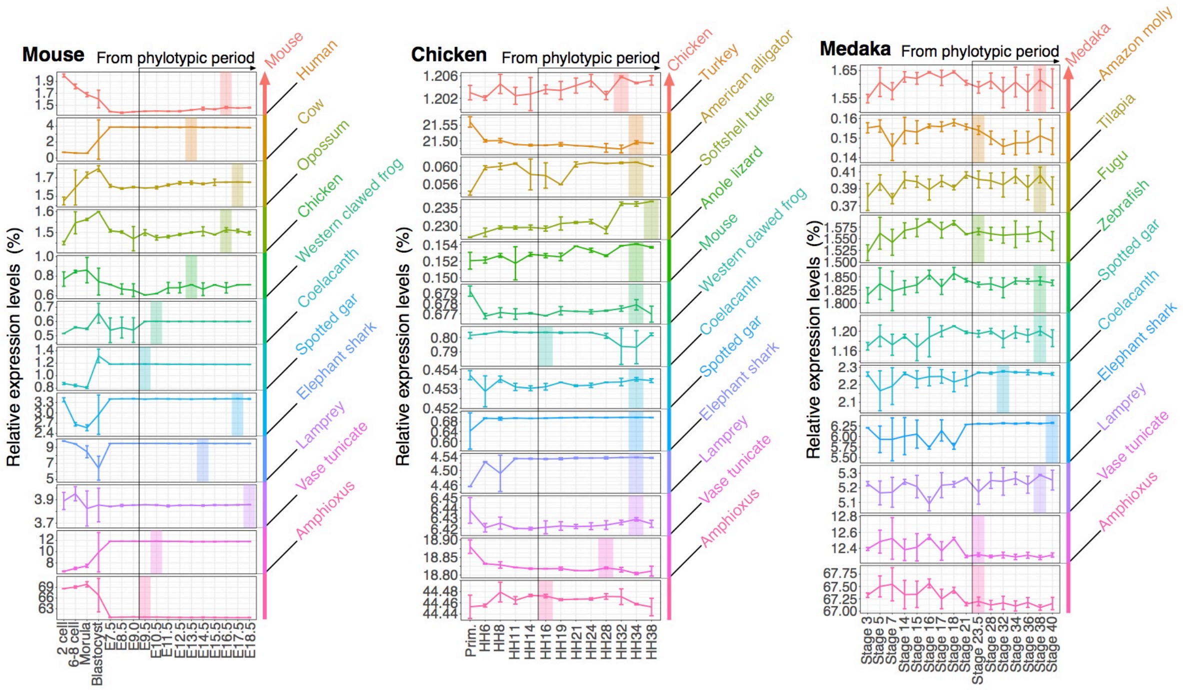

Figure S7. Developmental gene expression levels did not show a recapitulative pattern in the analysis including genes lost secondarily, related to Figure 4. The graphs presented were generated by using the same method as for Figure 4, except that the secondarily lost genes in any of the aligned species were included here (see Methods for details). In brief, the evolutionary age of each protein-coding gene was estimated according to the most recent common ancestors of all the species sharing the similar gene sequences. Statistical information of the Kruskal–Wallis rank sum test is given in Additional file 1: Tables S10.

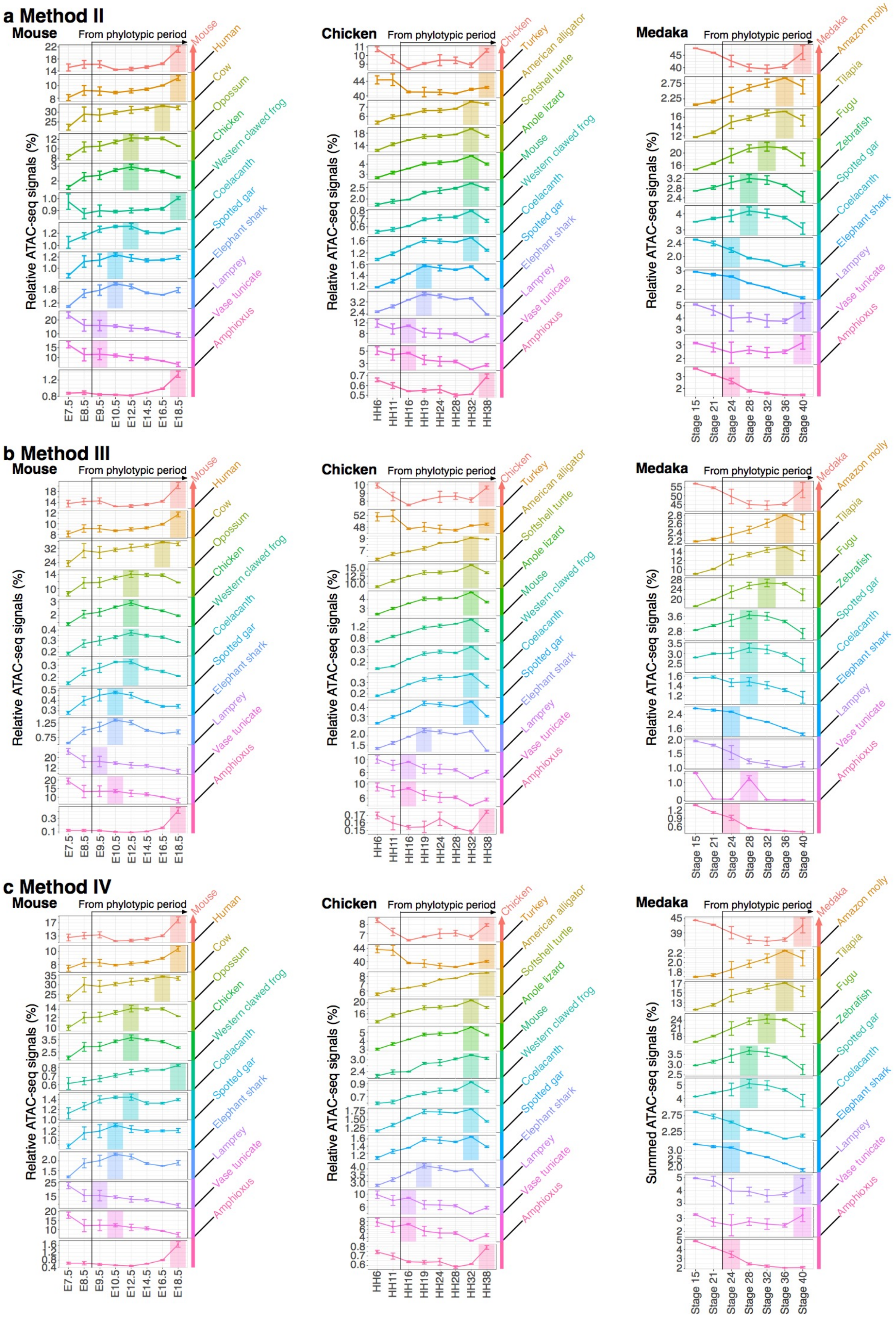

Figure S8. The recapitulative pattern of whole-embryo chromatin accessibility was consistent between methods I, II, III, and IV, related to Figure 3. **a**–**c** The graphs presented were generated by

using the same method as for Figure 3, except for the method to estimate the evolutionary ages of ACRs. In short, method II included ACRs that were estimated to be lost secondarily in any of the aligned species (**a**), whereas methods III and IV did not subdivide ACRs (see Methods, Additional file 3: Text S2.1, and Additional file 2: Figure S4 for details). For each ACR, we determined the species with similar sequences of the ACR, and then estimated the evolutionary age based on the most recent common ancestors of all these species. Method III excluded ACRs that were estimated to be lost secondarily in any of the aligned species (**b**), whereas method IV did not exclude these ACRs (**c**). Statistical information of the Kruskal–Wallis rank sum test is given in Additional file 1: Tables S11–13.

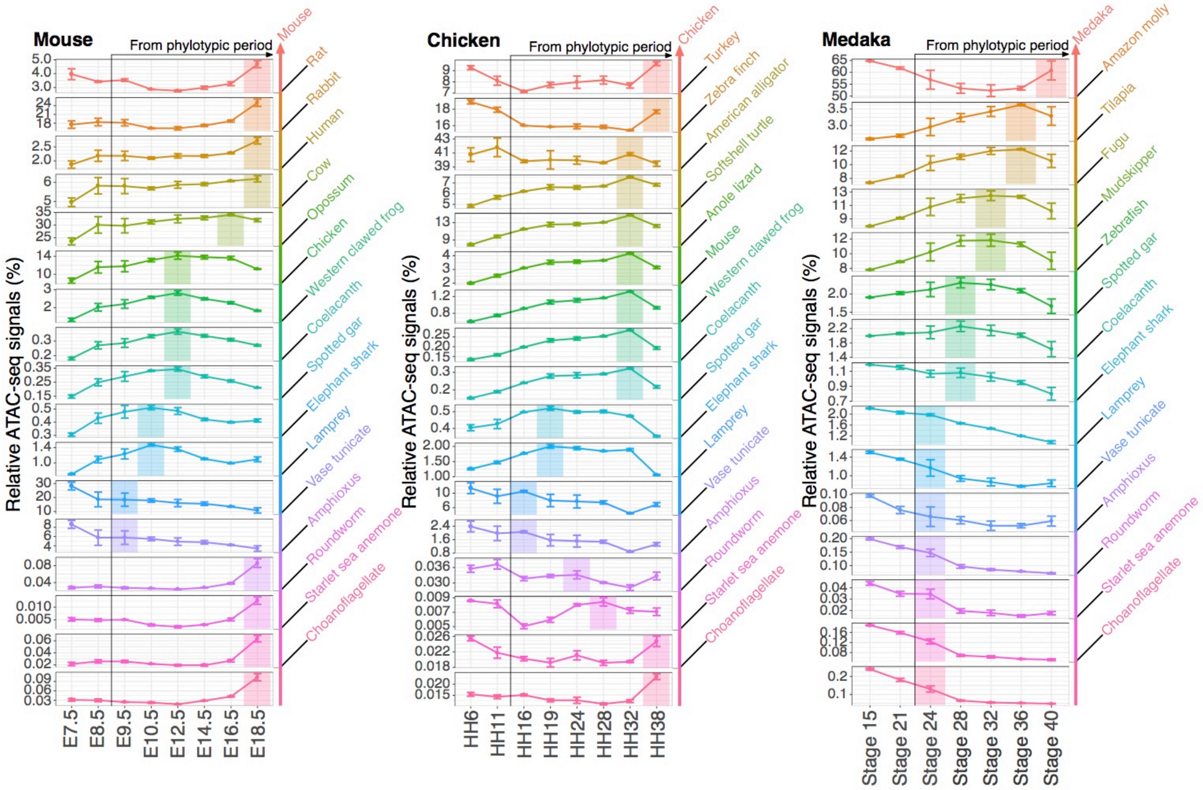

Figure S9. Essentially the same recapitulative pattern was observed in the analysis using different sets of species. The graphs were generated by using the same method as for Figure 3, except with different sets of genomes (see Additional file 3: Text S2.2 for details). Statistical information of the Kruskal–Wallis rank sum test is given in Additional file 1: Table S14.

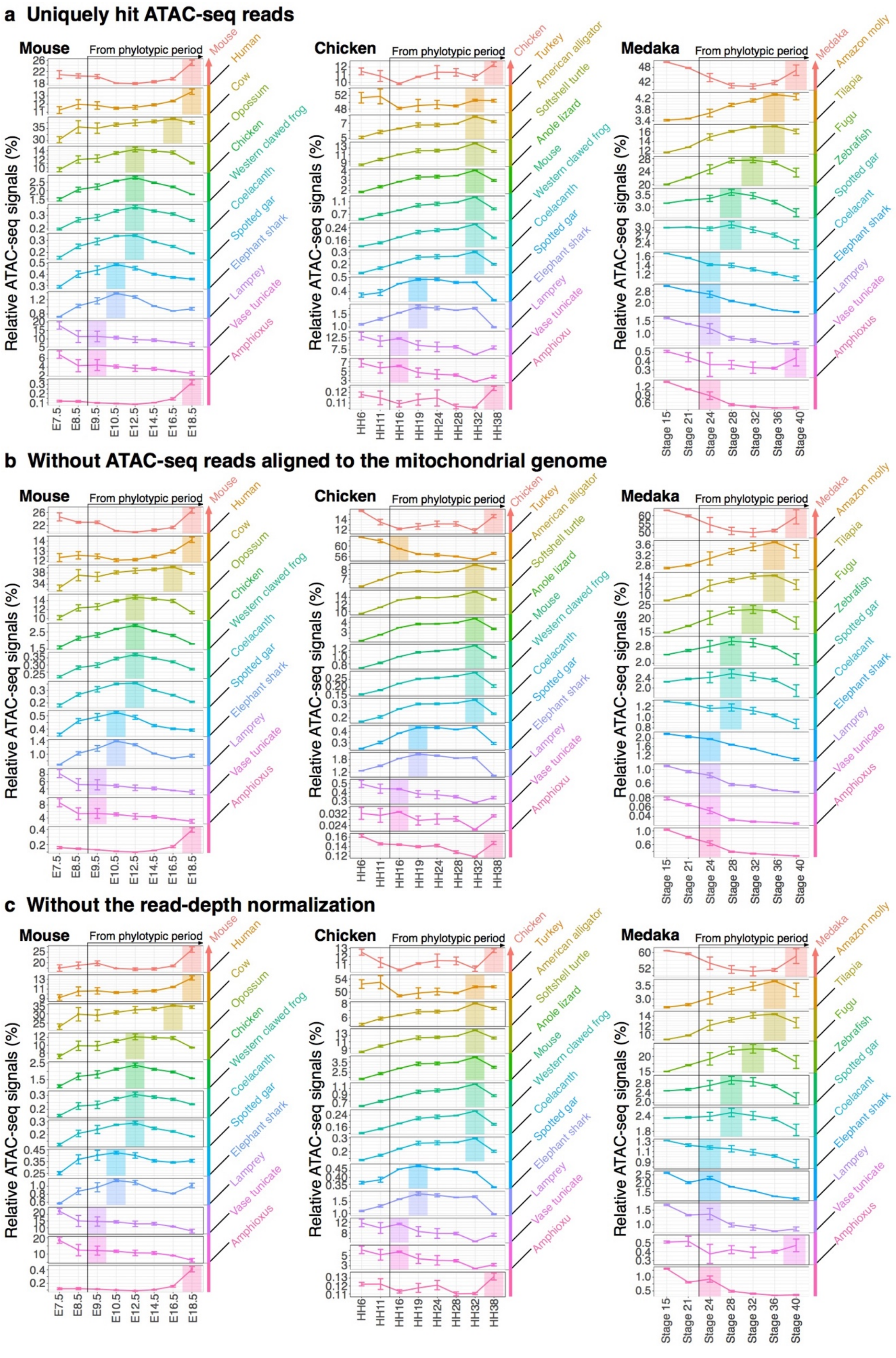

Figure S10. Essentially the same recapitulative pattern was observed for the analyses with different criteria in filtering ATAC-seq reads. The graphs were generated by using the same method as for

Figure 3, except that uniquely hit ATAC-seq reads were used (**a**; see Methods and Additional file 3: Text S2.3 for details), ATAC-seq reads aligned to the mitochondrial genome were removed (**b**; see Methods and Additional file 3: Text S2.4 for details), or aligned read depth was not normalized (**c**; see Methods and Additional file 3: Text S2.5 for details), respectively. Statistical information of the Kruskal–Wallis rank sum test is given in Additional file 1: Tables S15–17.

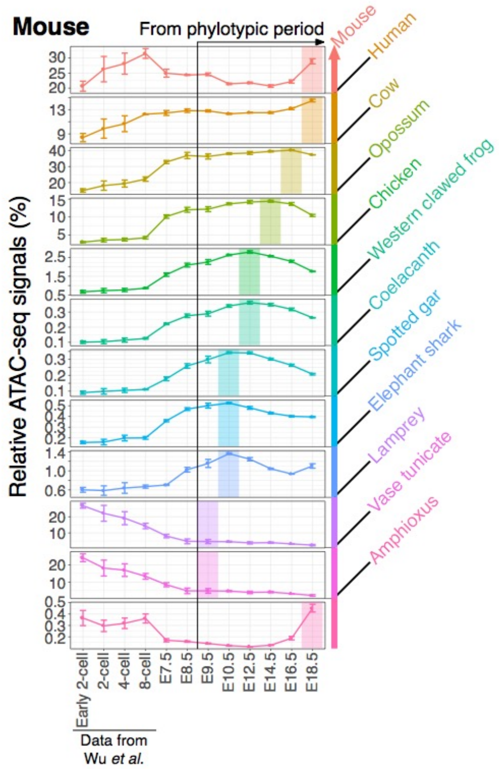

Figure S11. Chromatin accessibility of mouse early stages did not show the recapitulative pattern. Publicly available ATAC-seq data [28] of mouse preimplantation embryos (early two-cell stage, two-cell stage, four-cell stage, and eight-cell stage; see Methods for details) were used to test the recapitulative tendency in early mouse stages. The same method used for Figure 3 was applied for this analysis, except that ATAC-seq reads aligned to the mitochondrial genome were removed and aligned read depth was not normalized (see Methods for details). Statistical information of the Kruskal–Wallis rank sum test is given in Additional file 1: Table S18.

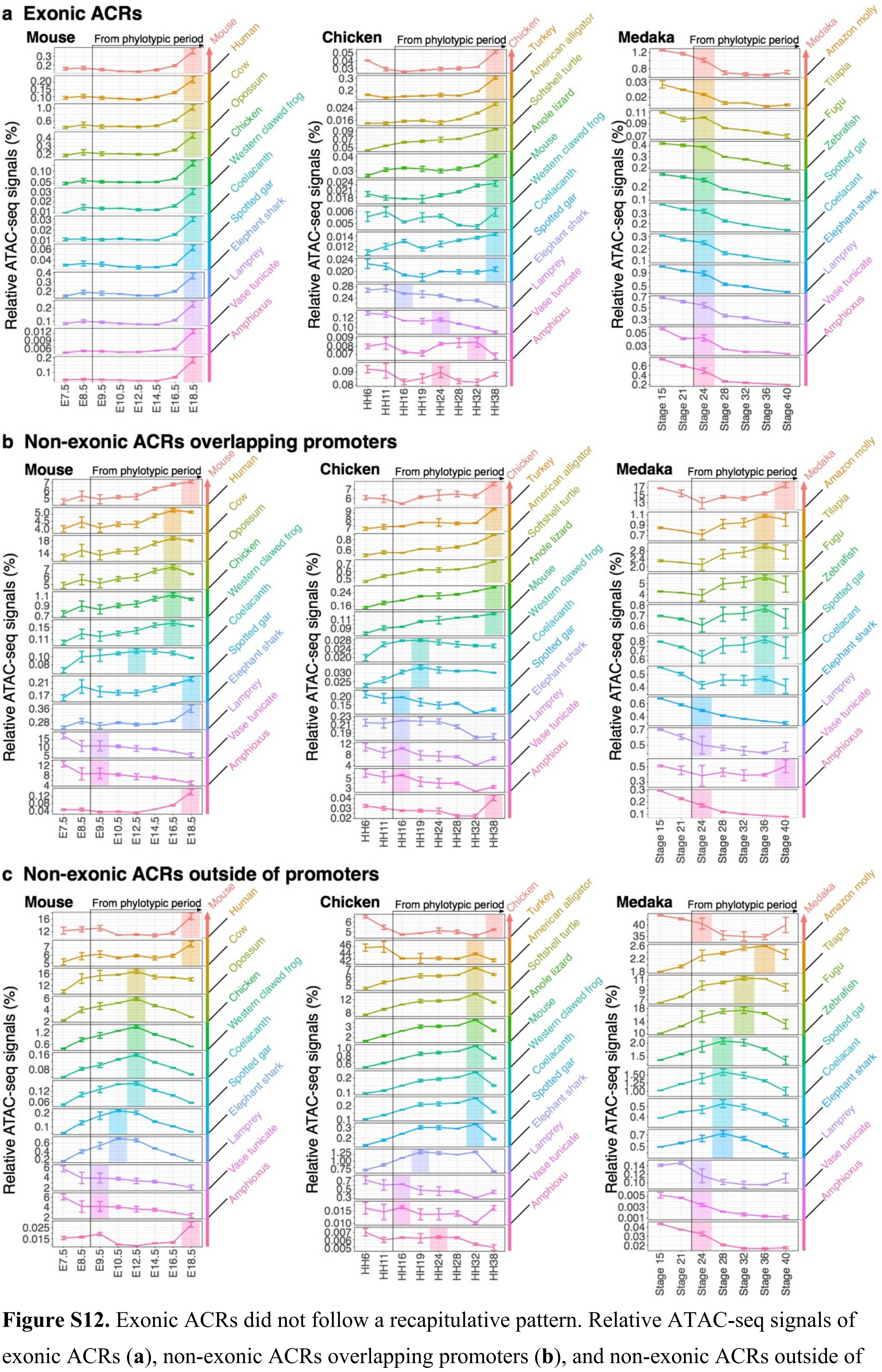

Figure S12. Exonic ACRs did not follow a recapitulative pattern. Relative ATAC-seq signals of exonic ACRs (**a**), non-exonic ACRs overlapping promoters (**b**), and non-exonic ACRs outside of

promoters (**c**) are shown. Note that relative ATAC-seq signals of non-exonic ACRs tended to show the recapitulative pattern. Statistical information of the Kruskal–Wallis rank sum test is given in Additional file 1: Tables S19–21.

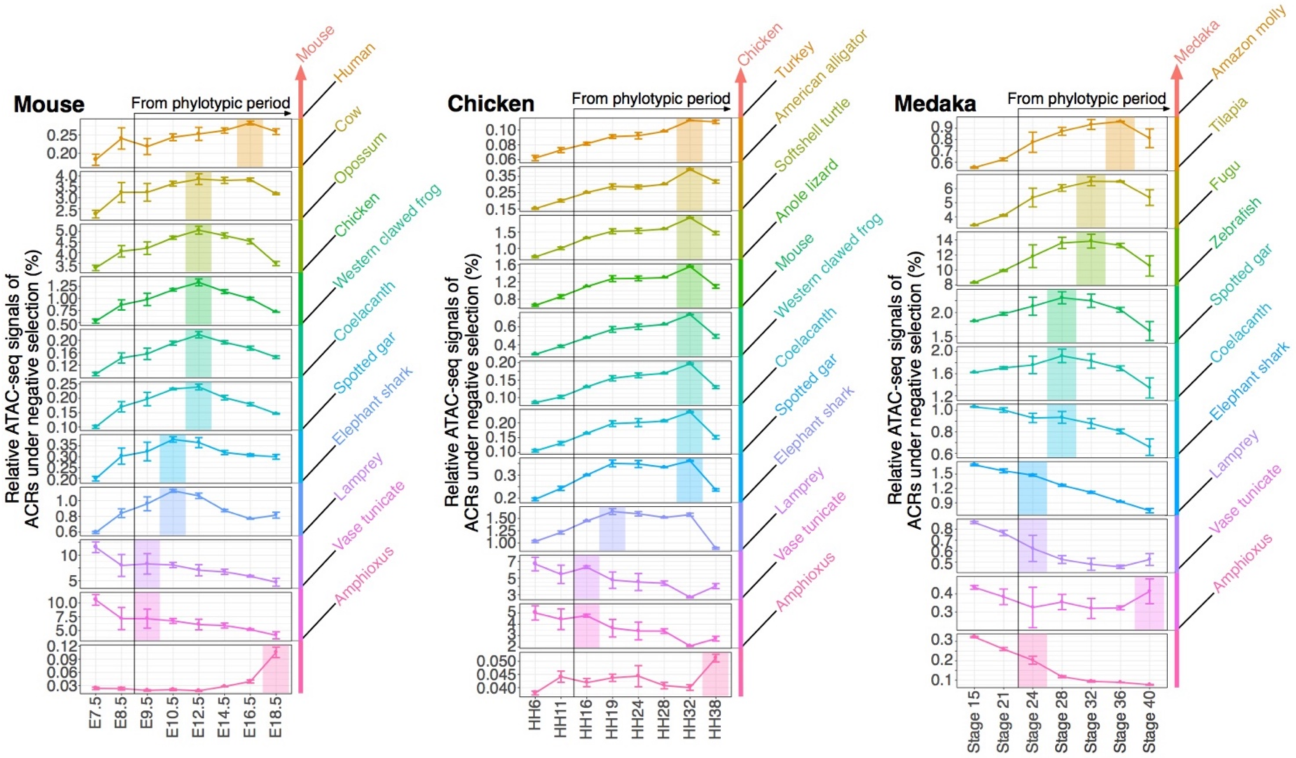

Figure S13. Similar recapitulative pattern observed for the chromatin accessibility under strong negative selection. The percentages corresponding to the summed signal intensity of regions under strong negative selection within ACRs (defined by phastCons [30]) for each evolutionary category divided by the total signal intensity for all ACRs in all categories at each developmental stage. Note that there is no graph for the evolutionary category of the newest ACRs, as the regions under the negative selection detected by phastCons are required to be aligned against one or more other species' genome(s), and species-specific ones are not included. Statistical information of the Kruskal–Wallis rank sum test is in Additional file 1: Table S22.

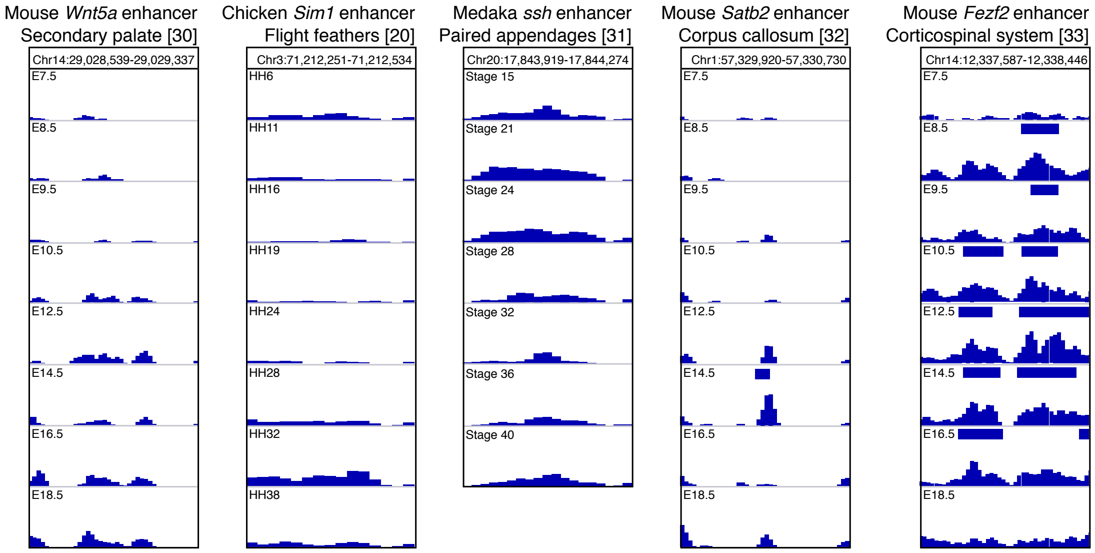

Figure S14. No ACR could be detected at three of five representative regulatory regions associated with taxon-specific features. These regulatory elements are known to be involved in the development of taxon-specific morphological features [20,31-34]. The mean ATAC-seq read enrichments of the three biological replicates (deep blue signals) and ACRs (deep blue boxes) at the five regulatory regions are shown. The two mouse enhancer regions overlapped identified ACRs, whereas no ACR could be detected at the remaining three enhancer regions by whole-embryo ATAC-seq analysis. This is consistent with previous studies showing that these enhancer activities were observed in a limited part of developing embryos (the frontonasal region for the mouse *Wnt5a* enhancer [31]; the posterior margin of the forelimb for the chicken *Sim1* enhancer [20]; the posterior regions of the pectoral fin buds for the medaka *shh* enhancer [32]). On the other hand, ACRs were detected at the *Satb2* and *Fezf2* enhancer regions in mouse embryos, possibly because these enhancer regions were accessible in a sufficient number of cells to be detected by whole-embryo ATAC-seq analysis (the deep layer of the neocortex for the mouse *Satb2* enhancer [33]; the neocortex for the mouse *Fezf2* enhancer [34]).
