# Supplementary material for: Recapitulation-like developmental transitions of chromatin accessibility in vertebrates
Source: Zoological Lett. 2019 Nov 14;5:33. doi: 10.1186/s40851-019-0148-9 (PMC6857340; doi:10.1186/s40851-019-0148-9)
Supplement: Supplementary file 3 — Additional file 3 Text S1. The phylotypic period in medaka embryogenesis. Text S2. Testing the recapitulative pattern with the different datasets. [file 40851_2019_148_MOESM3_ESM.pdf]

Additional file 3: Supplementary Texts

Text S1. The phylotypic period in medaka embryogenesis.

For chicken and mouse, previous studies identified stages around HH16 in chicken and E9.0 in mouse as the most transcriptomically conserved stages among vertebrates [6-8], and these may correspond to the phylotypic period of vertebrates [9,14,15]. As for medaka, a recent study [27] showed the transcriptomically conserved stages in embryogenesis are stages 19–25, based on pairwise comparisons between two teleosts (zebrafish and medaka). Nevertheless, it remains unclear whether these stages in medaka also show the highest conservation when compared against a wider range of vertebrate species (to be defined as the phylotypic period).

We performed a cross-species transcriptome comparison across six vertebrate species, using a previously described method [6-8]. In this analysis, we used previously reported early to late whole-embryo transcriptome data of mouse (*M. musculus*), chicken (*G. gallus*), softshell turtle (*P. sinensis*), western clawed frog (*X. tropicalis*), zebrafish (*D. rerio*), and medaka (*O. latipes*) [7,8,94]. Pairwise comparisons of the gene expression profiles of 1:1 orthologues showed that medaka at around stage 24 tends to have increased expression similarity to other vertebrate embryos (Additional file 2: Figure S1a–e). The stage 24 we identified here matches the stage that shows the most similar anatomical pattern between medaka and zebrafish and has been proposed as a potential phylotypic period in medaka (around the pharyngula stage) [105]. In addition, this stage overlapped with the conserved stages in a previous study (stages 19–25) [27]. By calculating previously developed expression distances [8] (expDists) with 1:1 orthologues (6,038 orthologues in total), which considers the phylogenetic relationship (rather than simply calculating pairwise similarities between species), we further demonstrated that the stages around stage 24 are the period showing the highest conservation across vertebrate species in medaka embryogenesis. In short, among the most similar (lowest 1% of expDists) combinations of stages, stage 24 was the developmental stage most frequently observed in medaka (Additional file 2: Figure S1f, g).

Using 1:1 orthologues for the comparison might introduce bias, as previously acknowledged [8], because several orthologue counterparts were removed. We therefore used orthologue groups to calculate expDists to cover all genes in each species and thus remove bias in the gene repertoire. In brief, the method assigns a 0 expression level to the genes of species in which the genes have no orthologous counterparts and sums the expression levels of genes within the same orthologue group (in-paralogs). Using this orthologue group–based method, we obtained essentially the same result as the one using 1:1 orthologues (Additional file 2: Figure S1f, g). Taken together, these results suggest that medaka embryogenesis shows hourglass-like divergence and that the period around stage 24 is the potential phylotypic period in this species.

Text S2. Testing the recapitulative pattern with the different datasets.

To test whether the recapitulative pattern observed in Figure 3 can be corroborated by analyses with different conditions, we prepared five types of datasets, as follows.

2.1 Alternative methods with different conditions to estimate the evolutionary ages of ACRs

Determining the evolutionary ages of gene regulatory regions based on sequence similarity is still an active area of research. To ensure that the overall recapitulative pattern is robust against the methods to estimate the evolutionary ages of ACRs, we performed the same analyses using the evolutionary ages of ACRs defined by methods I–IV (Figure 3 and Additional file 2: Figure S8). These four methods differ with regard to whether the ACRs are subdivided into multiple regions with different evolutionary ages and treated as individual ACRs, and whether the secondarily lost ACRs are excluded (for details, see Methods and Additional file 2: Figure S4). In all four methods, essentially the same recapitulative pattern was observed for all the examined species (Figure 3 and Additional file 2: Figure S8), except for those in the coelacanth–western clawed frog data in mouse.

2.2 Genome sets including more evolutionarily distant ones for estimating evolutionary ages of genomic regions

To study the evolutionary acquisition of regulatory regions during vertebrate evolution, we selected chordate species' genomes to estimate the evolutionary ages of genomic regions (Figure 3). However, it is unclear whether the recapitulative pattern would be similar to that in Figure 3 when more evolutionarily distant species were included. This analysis is also important for determining the evolutionarily older limit of the recapitulative pattern, especially for mouse and chicken embryogenesis. For these two species, using only the data shown in Figure 3, we cannot conclude that regions newer than the olfactore–cephalochordate split show sequential transitions of chromatin accessibility, because the second-oldest category of regions (the second from the bottom in each panel of Figure 3) contains not only the regions that emerged during evolution from the olfactore–cephalochordate split to the vertebrate–urochordate split, but also older regions that have been lost from the cephalochordate lineage. In contrast, as with medaka, we can conclude that the transitional pattern of whole-embryo chromatin accessibility of ACRs newer than the vertebrate–urochordate split shows a recapitulative pattern in Figure 3, because the signal peaks of the second-oldest category did not follow the pattern. Therefore, we performed the same analysis but with genomes of different apoikozoan (consisting of the Animalia and the Choanoflagellatea) species. For mouse and chicken embryogenesis, we noted the recapitulative pattern of whole-embryo chromatin accessibility, similar to that shown in Figure 3, and found that the evolutionary ages of ACRs that tend to follow this pattern were newer than the olfactore–cephalochordate split (Additional file 2: Figure S9). In contrast, regarding medaka, although the similar pattern was observed in later embryogenesis, the older limit of the evolutionary range for this pattern differed depending on the genome set used for this analysis (Figure 3 and Additional

file 2: Figure S9). However, the chromatin accessibility of ACRs newer than the vertebrate–urochordate split consistently followed the recapitulative pattern in these two analyses, indicating that this pattern itself was robust against the genome set used.

2.3 Uniquely hit ATAC-seq reads

Because some ATAC-seq reads aligned to multiple genomic regions (e.g., transposable elements [106], which have many copies in the genome), we used the best-hit genomic region for the analysis in Figure 3. To test whether this best-hit parameter biased the result, we prepared another dataset made of uniquely hit ATAC-seq reads, which excluded ATAC-seq reads that were aligned to multiple regions in the genome. This dataset yielded essentially the same results as in Figure 3 (Additional file 2: Figure S10a).

2.4 Without ATAC-seq reads aligned to the mitochondrial genome

ATAC-seq reads can contain various proportions of sequences from mitochondrial genomes [47]. In our ATAC-seq data, the average mitochondrial fractions were 8.7%, 7.7%, and 2.9% of properly aligned reads (duplicate reads not included) in mouse, chicken, and medaka embryos, respectively. Because these percentages were low compared with those in other studies using ATAC-seq without any treatment for mitochondrial DNA depletion (between 10% and >50% [47]; between 30% and 50% [107]; >80% [28]; between 20% and 80% [108]), we did not exclude ATAC-seq reads aligned to the mitochondrial genome to perform the analysis shown in Figure 3. However, we cannot exclude the possibility that including these fractions influenced the observed recapitulative pattern. We therefore removed ATAC-seq reads that were aligned to the mitochondrial genome and assessed whether the recapitulative pattern was still apparent. For chicken, although the developmental stage with the maximum chromatin accessibility in the second-newest category changed from HH38 to HH16 (Additional file 2: Figure S10b), the overall pattern was essentially the same as that in Figure 3. For medaka, although including mitochondrial sequences altered the older limit of the evolutionary range over the recapitulative pattern during late embryogenesis (Additional file 2: Figure S10b), we observed the similar pattern for regions newer than the vertebrate–urochordate split.

2.5 Without the read-depth normalization

In order to avoid potential biases arising from the different ATAC-seq read depths among samples, we performed read-depth normalization by randomly selecting 20 million reads of genome-mapped reads (see Methods for details; Figure 3). It is also possible that this read-depth normalization could influence the observed recapitulative pattern. To test this possibility, we performed the same analysis without the read-depth normalization. Essentially the same results were obtained (Additional file 2: Figure S10c), indicating that this random selection of reads did not affect our conclusion.

References only in Supplementary Texts

105. Tena JJ, González-Aguilera C, Fernández-Miñán A, Vázquez-Marín J, Parra-Acero H, Cross JW, et al. Comparative epigenomics in distantly related teleost species identifies conserved cis-regulatory nodes active during the vertebrate phylotypic period. Genome Res. 2014;24:1075–85.

106. Feschotte C. Transposable elements and the evolution of regulatory networks. Nat. Rev. Genet. 2008;9:397–405.

107. Sos BC, Fung H-L, Gao DR, Osothprarop TF, Kia A, He MM, et al. Characterization of chromatin accessibility with a transposome hypersensitive sites sequencing (THS-seq) assay. Genome Biol. 2016;17:20.

108. Montefiori L, Hernandez L, Zhang Z, Gilad Y, Ober C, Crawford G, et al. Reducing mitochondrial reads in ATAC-seq using CRISPR/Cas9. Sci Rep 2017;7:2451.
